# Genomic insights into the critically endangered King Island scrubtit

**DOI:** 10.1093/jhered/esae029

**Published:** 2024-05-30

**Authors:** Ross Crates, Brenton von Takach, Catherine M Young, Dejan Stojanovic, Linda E Neaves, Liam Murphy, Daniel Gautschi, Carolyn J Hogg, Robert Heinsohn, Phil Bell, Katherine A Farquharson

**Affiliations:** Fenner School of Environment and Society, Australian National University, Canberra 2601, Australia; School of Molecular and Life Sciences, Curtin University, Perth 6845, Australia; Fenner School of Environment and Society, Australian National University, Canberra 2601, Australia; Fenner School of Environment and Society, Australian National University, Canberra 2601, Australia; Fenner School of Environment and Society, Australian National University, Canberra 2601, Australia; Fenner School of Environment and Society, Australian National University, Canberra 2601, Australia; Fenner School of Environment and Society, Australian National University, Canberra 2601, Australia; School of Life and Environmental Sciences, The University of Sydney, Sydney 2050, Australia; Australian Research Council Centre of Excellence for Innovations in Peptide and Protein Science, The University of Sydney, Sydney 2050, Australia; Fenner School of Environment and Society, Australian National University, Canberra 2601, Australia; Biological Sciences, School of Natural Sciences, University of Tasmania, Hobart, Tasmania 7005, Australia; School of Life and Environmental Sciences, The University of Sydney, Sydney 2050, Australia; Australian Research Council Centre of Excellence for Innovations in Peptide and Protein Science, The University of Sydney, Sydney 2050, Australia

**Keywords:** Australia, conservation genetics, genetic rescue, ornithology, population monitoring, translocation

## Abstract

Small, fragmented, or isolated populations are at risk of population decline due to fitness costs associated with inbreeding and genetic drift. The King Island scrubtit *Acanthornis magna greeniana* is a critically endangered subspecies of the nominate Tasmanian scrubtit *A. m. magna,* with an estimated population of <100 individuals persisting in three patches of swamp forest. The Tasmanian scrubtit is widespread in wet forests on mainland Tasmania. We sequenced the scrubtit genome using PacBio HiFi and undertook a population genomic study of the King Island and Tasmanian scrubtits using a double-digest restriction site-associated DNA (ddRAD) dataset of 5,239 SNP loci. The genome was 1.48 Gb long, comprising 1,518 contigs with an N50 of 7.715 Mb. King Island scrubtits formed one of four overall genetic clusters, but separated into three distinct subpopulations when analyzed independently of the Tasmanian scrubtit. Pairwise *F*_ST_ values were greater among the King Island scrubtit subpopulations than among most Tasmanian scrubtit subpopulations. Genetic diversity was lower and inbreeding coefficients were higher in the King Island scrubtit than all except one of the Tasmanian scrubtit subpopulations. We observed crown baldness in 8/15 King Island scrubtits, but 0/55 Tasmanian scrubtits. Six loci were significantly associated with baldness, including one within the DOCK11 gene which is linked to early feather development. Contemporary gene flow between King Island scrubtit subpopulations is unlikely, with further field monitoring required to quantify the fitness consequences of its small population size, low genetic diversity, and high inbreeding. Evidence-based conservation actions can then be implemented before the taxon goes extinct.

## Introduction

When populations decline and become fragmented, changes to patterns of gene flow and reductions in effective population size can exacerbate population declines through depressed individual fitness associated with inbreeding and genetic drift (Frankham 1995). Therefore, the importance of understanding the dynamics of threatened species’ populations at the molecular level is increasingly being acknowledged in conservation (Allendorf et al. 2010). Without such knowledge, potentially significant drivers of population decline can be overlooked if the focus of monitoring efforts is solely at the individual or population level (Stojanovic et al. 2022).

Although there is minimal risk of outbreeding depression, genetic rescue of small, inbred populations can have significant conservation benefits (Frankham et al. 2011, 2015). Meta-analysis revealed that outcrossing through the introduction of novel genes from external populations had beneficial effects on over 90% of inbred populations examined, with a median increase in composite fitness of over 145% in stressful environments (Frankham et al. 2015). Examples of successful genetic rescue include the mountain pygmy possum *Burramys parvus*, whose population more than doubled in 3 years following the introduction of six males from an external population (Weeks et al. 2017), and the bighorn sheep *Ovis canadensis*, whereby experimental restoration of immigration into a small inbred population led to increases in fitness-related traits of 23% to 257% (Hogg et al. 2006).

Here we address a key knowledge gap in our understanding of the critically endangered King Island scrubtit *Acanthornis magna greeniana* (Geyle et al. 2018). The King Island scrubtit is a subspecies restricted to King Island, a 1,098 km^2^ island within the Bass Strait, between Victoria and mainland Tasmania. The nominate Tasmanian scrubtit (*A. m. magna*) occurs on mainland Tasmania. Formal survey of researchers in the field of conservation biology estimated that the King Island scrubtit had an 83% probability of extinction within 20 years (95% CI = 66% to 93 %, Geyle et al. 2018). The primary driver of King Island scrubtit population decline is habitat loss (Webb et al. 2016). While the exact habitat preferences of King Island scrubtits are not certain, surveys suggest the birds prefer swamp forest containing dominant or subdominant swamp paperbarks *Melaleuca ericifolia* (Webb et al. 2016; Bell et al. 2023). It is likely that the King Island scrubtit was widespread in suitable native vegetation prior to European colonization >100 years ago, but extensive surveys of potentially suitable habitat fragments suggest it is now confined to only three patches: Colliers Swamp in the south, Pegarah State Forest and surrounding forests in the east, and Lavinia State Reserve and a small area of private land between The Nook swamps and Granite Lagoon in the northeast (Webb et al. 2016; Bell et al. 2023, Fig. 1). The total estimated area of occupancy of the King Island scrubtit is <1 km^2^ and likely declining (Webb et al. 2016).

**Fig. 1.**
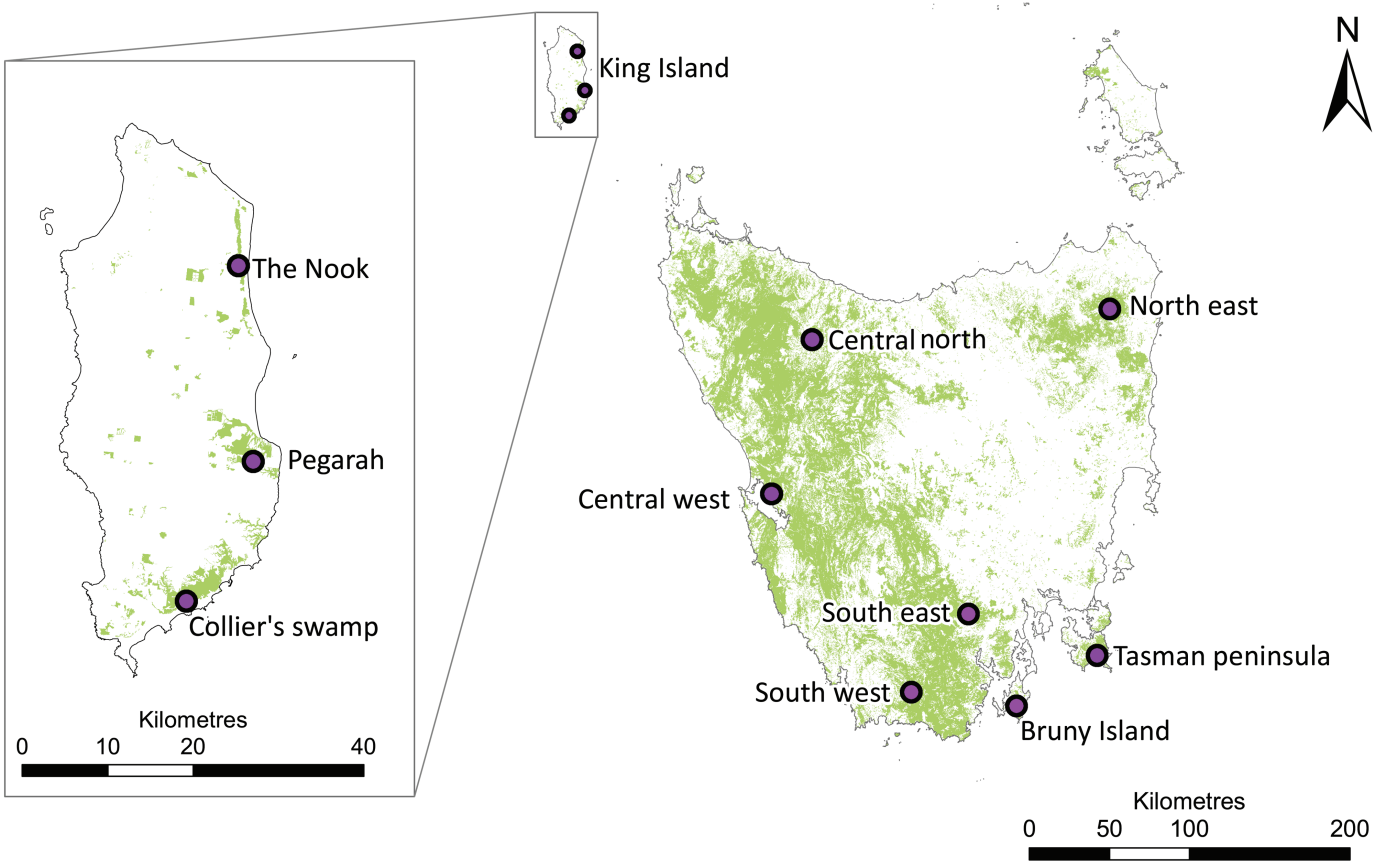
Sampling locations of Tasmanian scrubtit (right) and King Island scrubtit (left). Shading shows the distribution of wet eucalypt forest, swamp forest, and rainforest. Vegetation data are derived from TASVEG v4.0.

The three known putative subpopulations of the King Island scrubtit are separated by 18 to 20 km, between which lies a matrix dominated by agricultural land, sand dunes, and potentially unsuitable scrub and heath vegetation types (Fig. 1). The extent to which individual scrubtits can permeate the matrix and facilitate gene flow between the subpopulations is unknown, but we expect it to be low based on the species’ habitat preferences. Field surveys estimate the Colliers Swamp subpopulation contains approximately 30 to 40 birds, Pegarah Forest 15 to 30 birds, and Nook Swamps 10 to 20 birds (Webb et al. 2016; Bell et al. 2023). There is a risk that genomic impacts associated with small effective population size and limited dispersal capacity may be contributing to the decline of the King Island scrubtit population (Frankham 2015). Other threats facing King Island scrubtits include acid sulfate soils associated with drainage for agriculture, wildfires, predation by feral cats, and habitat deterioration through wind throw and sea-level rise (Webb et al. 2016; Fielding et al. 2022). The King Island scrubtit is therefore a priority species for conservation actions under the Australian Government’s Threatened Species Action Plan 2022–2032 (Commonwealth of Australia 2022).

In contrast to the King Island scrubtit, Tasmanian scrubtits are not considered threatened and are widespread in suitable habitats on mainland Tasmania and close offshore islands, though there is currently no reliable population estimate (BirdLife International 2022). The species prefers wet forest habitats which dominate the western side of mainland Tasmania, but are patchier on the eastern side (Fig. 1). There is no quantitative data on dispersal in Tasmanian or King Island scrubtits, but the species is understood to be largely sedentary (Higgins et al. 2006).

To assess the need for and to inform potential genetic management of the King Island scrubtit, our study had four aims: first to sequence the Tasmanian scrubtit genome; second to estimate the current spatial genetic structure of the King Island scrubtit population; third to quantify current levels of genetic diversity, inbreeding, and relatedness within the King Island scrubtit population; and finally to consider the population genetics of the King Island scrubtit within the context of the genetics of the Tasmanian scrubtit. Following population genetic theory and available demographic data (Frankham 1995; Webb et al. 2016), we predicted that, relative to the Tasmanian scrubtit, the King Island scrubtit would have clearer population genomic structuring, higher levels of inbreeding, and less genetic diversity.

## Materials and methods

### Sample collection

To obtain indicative genetic diversity metrics across mainland Tasmania, we sampled between 5 and 11 scrubtits from seven a priori subpopulations on mainland Tasmania (including Bruny Island) during the nonbreeding season (January to March 2021). Due to small population sizes and licensing restrictions on King Island, we sampled five individuals from each of the three locations during the same nonbreeding season (Table 1, Fig. 1). We trapped scrubtits using a single 6 m mist net and 1 min of scrubtit song broadcast using portable speakers (ANU animal ethics permit # A2021/33). We sampled blood (<20 μl per individual) using the standard brachial venepuncture technique with a 0.7 mm needle into 70% ethanol. For two individuals from whom we were unable to safely obtain blood, we collected feathers shed during handling. One male Tasmanian scrubtit was collected under licence (see acknowledgements) for genome sequencing, from which organ tissue samples (heart, spleen, kidney, gonads, brain, and liver) were taken (Table S1). For each individual we took standard morphometric measurements and scanned for any unusual physical features such as feather abnormalities or skin lesions that may be indicators of poor health. A single observer (CY) sampled and measured all birds, and the maximum capture time was 35 min. No birds showed adverse reactions to sampling and all flew off strongly upon release. The 15 individuals sampled on King Island was the maximum permissible sample size under licence conditions.

**Table 1.** Sample sizes by molecular sex (F/M) and population genetic parameters for the King Island and Tasmanian scrubtit. Parameters shown are number of alleles (A), effective number of alleles (A_E_), SNP expected heterozygosity (H_E_), SNP observed heterozygosity (H_O_), mean individual inbreeding coefficient (II_C_), allelic richness (A_R_), mean private alleles (PA), and within population mean kinship (MK mean ± SE) for King Island populations only, and for all subpopulations. See Table S6 for estimates with standard errors.

Region	Subpopulation	#F	#M^a^	A	A_E_	H_E_	H_O_	II_C_	A_R_	P_A_^b^	MK (KI only)	MK (all subpops)
King Island	Colliers Swamp	1	4	1.423	1.258	0.169	0.17	0.321	1.343	45.69 (215)	0.0968 ± 0.0167	0.2658 ± 0.0095
	Nook	2	3	1.489	1.302	0.197	0.2	0.234	1.401	39.28 (383)	0.0431 ± 0.0265	0.2094 ± 0.0172
	Pegarah	2	3	1.448	1.277	0.181	0.166	0.349	1.367	46.77 (252)	0.0671 ± 0.0353	0.2273 ± 0.0238
Mainland	Central North	3	7	1.816	1.449	0.282	0.27	0.087	1.597	41.33		0.0331 ± 0.0034
	Central West	3	3	1.753	1.439	0.284	0.27	0.084	1.588	32.99		0.0254 ± 0.0015
	North East	3	4	1.616	1.343	0.22	0.216	0.192	1.462	39.05		0.1543 ± 0.0015
	South Bruny Island	3	4	1.671	1.394	0.251	0.243	0.169	1.521	84.16		0.1211 ± 0.0022
	South East	2	9	1.838	1.449	0.282	0.27	0.077	1.601	57.85		0.0231 ± 0.0035
	South West	2	3	1.713	1.426	0.28	0.268	0.082	1.572	29.09		0.0272 ± 0.0018
	Tasman Peninsula	3	5	1.448	1.266	0.167	0.163	0.346	1.346	65.79		0.2650 ± 0.0014

^a^1 additional male collected from Weilangta for genome sequencing.

^b^Numbers in parentheses denote number of private alleles within the King Island subpopulations when analyzed separately.

### DNA extraction, sexing, and sequencing

High molecular weight DNA was extracted from flash frozen heart and kidney using the Nanobind Tissue Big DNA Kit v1.0 11/19 (Circulomics). A Qubit fluorometer (Thermo Fisher Scientific) was used to quantify DNA concentrations with the Qubit dsDNA BR assay kit (Thermo Fisher Scientific). RNA was extracted from heart, spleen, kidney, gonads, brain, and liver stored in RNA later using the RNeasy Plus mini Kit (Qiagen) with RNAse-free DNAse (Qiagen) digestion. RNA quality was assessed via Nanodrop (Thermo Fisher Scientific). We extracted DNA for population genomics from blood and feather samples using the Monarch Genomic DNA Purification Kit (New England BioLabs, Victoria, Australia). We quantified DNA concentrations using a Qubit 3.0 fluorometer (yield range 10.3 to 209 ng μl^−1^, Table S1) and standardized the concentration of each sample to 10 to 30 ng µl^−1^ DNA for 20 to 25 μl and determined the sex of individuals using a polymerase chain reaction (PCR) protocol adapted from Fridolfsson and Ellegren (1999, Supplementary File S1). We arranged the samples on a single 96 well plate, containing five technical replicates of the samples with the highest DNA concentrations, an additional 21 nontechnical replicates including all of the King Island samples, five extra samples from mainland Tasmania, and one negative control.

Double-digest restriction associated DNA (ddRAD) sequencing following Peterson et al. (2012) was undertaken at the Australian Genome Research Facility, Melbourne on an Illumina NovaSeq 6000 platform using 150 bp paired-end reads. Samples were first quantified using Quantifluor and visualized on 1% agarose e-gel to ensure all samples exceeded the minimum input DNA quantity of 50 ng. Three establishment samples with at least 250 ng DNA that were representative of the distribution of the samples (2 Tasmanian scrubtits and 1 King Island scrubtit) were used to determine the optimal combination of restriction enzymes, which were *EcoRI* and *HpyCH4IV*. Further details on the library preparation protocol are provided in Supplementary File S1.

### Genome sequencing and assembly

Full methodological details of the genome and transcriptome sequencing and assembly are provided in Supplementary File S2. In summary, high molecular weight DNA was sent for PacBio HiFi library preparation with Pippin Prep and sequencing on one single molecule real-time (SMRT) cell of the PacBio Sequel II (Australian Genome Research Facility, Brisbane, Australia). Total RNA was sequenced as 100 bp paired-end reads using Illumina NovaSeq 6000 with Illumina Stranded mRNA library preparation at the Ramaciotti Centre for Genomics (University of New South Wales, Sydney, Australia). Genome assembly was conducted on Galaxy Australia (The Galaxy Community 2022) following the genome assembly guide (Price and Farquharson 2022) using HiFiasm v0.16.1 with default parameters (Cheng et al. 2021, 2022). Transcriptome assembly was conducted on the University of Sydney High Performance Computer, Artemis. Genome annotation was performed using FGENESH++ v7.2.2 (Softberry; (Solovyev et al. 2006)) on a Pawsey Supercomputing Centre Nimbus cloud machine (256 GB RAM, 64 vCPU, 3 TB storage) using the longest open reading frame predicted from the global transcriptome, nonmammalian settings, and optimized parameters supplied with the *Corvus brachyrhynchos* (American crow) gene-finding matrix. The mitochondrial genome was assembled using MitoHifi v3 (Uliano-Silva et al. 2023). Benchmarking universal single-copy orthologs (BUSCO) was used to assess genome, transcriptome, and annotation completeness (Manni et al. 2021).

### Bioinformatics pipeline and SNP filtering

Raw sequence data were processed using Stacks v2.62 (Catchen et al. 2013) and aligned to the genome with BWA v0.7.17-r1188 (Li and Durbin 2009). Full details of the bioinformatics pipeline, which produced a variant call format (VCF) file containing 45,488 variants for SNP filtering in R v4.0.3 (R Core team 2020) are provided in Supplementary File S1. We filtered genotyped variants using the “SNPfiltR” v1.0.0 package (DeRaad 2022) based on 1) minimum read depth (≥5), 2) genotype quality (≥20), 3) maximum read depth (≤137), and 4) allele balance ratio (0.2 to 0.8). Then, using a custom R script, we filtered SNPs based on 1) the level of missing data (<5%); 2) minor allele count (MAC ≥3), 3) observed heterozygosity (<0.6), and 4) linkage disequilibrium (correlation <0.5 between loci within 500,000 bp).

To ensure that relationships between individuals could be accurately inferred from the data, we used these SNPs and samples to construct a hierarchical clustering dendrogram based on genetic distance, with visual examination of the dendrogram confirming that all 24 replicates paired closely together on long branches (Fig. S1). The percentage difference between called genotypes of technical replicates was also used to confirm that genotyping error rates were low after filtering (mean 99.91% ± 0.005% SE similarity between replicates). We therefore removed one of each replicate pair from all further analyses. We also made a higher-level bootstrapped dendrogram by using genetic distances among sampling localities instead of individuals (Fig. S2).

We used “tess3r” (Caye et al. 2016, 2018) to perform a genome scan for loci under selection, using the Bejamini–Hochberg algorithm (Benjamini and Hochberg 1995), with a false discovery rate of 1 in 10,000 to correct for multiple testing. Because this method identified zero candidate loci under selection, we also used the *gl.outflank* function in “dartR” v2.0.4 to implement the OutFLANK method (Whitlock and Letterhos 2015) to infer the distribution of *F*_ST_ for loci unlikely to be strongly affected by spatially diversifying selection. This method also identified zero putatively adaptive loci, leaving a final dataset for formal population genetic analysis containing all 70 originally sampled individuals, 5,239 biallelic SNPs, and an overall missing data level of 0.98%. The number of SNPs and samples removed from the dataset at each filtering step is provided in Table S2.

### Statistical analysis

#### Population genomic structure.

We used “poppr” v2.9.3 (Kamvar 2023) to produce a multidimensional scaling (MDS) plot of all individuals based on a matrix of Prevosti’s genetic distances. To focus in more detail on the King Island population, we then repeated the MDS analysis using only the 15 individuals sampled from King Island. We then created a pairwise Weir and Cockerham (1984) fixation index (*F*_ST_) matrix in “StAMPP” v1.6.3 (Pembleton 2013) with 999 bootstraps to assess significance of the estimated differentiation between populations. To assess isolation by distance at the individual level, we fitted a linear model of all standardized pairwise *F*_ST_ values against the geographic distance between samples (Rousset 1997).

We used the alternating projected least-squares algorithm implemented in “tess3r” to assign individuals to ancestral population genomic clusters, investigate patterns of admixture between populations, and assess hierarchical population structure. This method applies a model of genetic structure featuring a number of ancestral populations (*k*), allowing assessment of values for *k* that minimise cross-entropy values (Frichot et al. 2014). It also incorporates the spatial location of sampling, to remove potential bias associated with isolation by distance. We calculated cross-entropy criteria for values of *k* between 1 and 15, and visualized a cross-entropy scree-plot to identify a plateau or substantial change in curvature in the plot. We then extracted the matrices of individual admixture coefficients for the most relevant values of *k* for inference and plotted these as stacked bar plots to visualize hierarchical population structure. We then interpolated values of *k* from 2 to 4 across the landscape within the range of the scrubtit, based on the geographical location of samples. We then repeated this analysis using only the King Island scrubtit data for values of *k* from 1 to 3.

#### Genetic diversity, inbreeding and mean kinship.

We used the *genetic_diversity* function within the “gstudio” package v1.5.3 (Dyer 2021) to calculate mean (±standard error and standard deviation) genetic diversity metrics across the 10 subpopulations. These metrics included the number of alleles (A), effective number of alleles (A_E_), observed heterozygosity (H_O_), and expected heterozygosity (H_E_). We used the standard correction for small sample size to account for potential biases in the estimates of H_E_ due to variation in the number of samples obtained from subpopulations. We then used “PopGenReport” v3.0.7 (Gruber and Adamack 2022) to calculate mean allelic richness (A_R_) in the subpopulations and “poppr” to calculate 1) the mean number of private alleles within all subpopulations, bootstrapped to sample size of five, and 2) the number of private alleles only within subpopulations of the King Island scrubtit. Finally, we calculated individual inbreeding coefficients (II_C_) using the “mom.weir” method with the *snpgdsIndInbCoef* function in “SNPRelate” (Zheng et al. 2012). This method uses a modified Visscher’s estimator (Yang et al. 2010).

We calculated relatedness using COANCESTRY (Wang 2011), firstly only within and between the King Island subpopulations, and then within all 10 subpopulations together (King Island and mainland Tasmania). We first ran simulations as per Hogg et al. (2019) to determine the best moment estimator to use. This was the triadic likelihood method (TrioML), as it weights loci relative to the number of alleles and accounts for genotyping error and inbreeding (Wang 2007). Results are presented as mean kinship values (MK), calculated by dividing the TrioML value by two.

#### Genetic relationship with crown baldness.

During sampling, we noted that eight King Island scrubtits had a distinct bald patch on the crown of their heads (Fig. S3), so explored the possibility that this feature was linked to genome-wide heterozygosity or particular SNP loci in these individuals. We used “inbreedR” v 0.3.3 (Stoffel et al. 2016) to calculate multi-locus heterozygosity (MLH) values for each individual, then implemented a heterozygosity-fitness correlation analysis using logistic regression via package “lme4” v1.1-31 (Bates et al. 2015) with a binomial response of bald or not bald against for the King Island scrubtits sampled. We included genotypic sex as a random term.

To determine if any loci were significantly associated with baldness, we used a latent factor mixed modeling (LFMM) approach. This method tests the explanatory significance of a trait variable on the genotypic matrix, allowing for inference regarding the genetic basis of the trait. As LFMM requires no missing data, we imputed missing genotypes via the “impute” function of the “LEA” package, utilizing four ancestral populations and method = “mode.” We then used the “lfmm2” exact least-squares function of the LEA package to build the LFMM object and identified allele frequencies that were correlated with each of the environmental variables (Caye et al. 2019). This method controls for population structure via a number of latent factors equal to the number of ancestral populations. We adjusted the *P*-values for each SNP using the robust estimate of the genomic inflation factor (Martins et al. 2016) and a Benjamini–Hochberg correction (Benjamini and Hochberg 1995) to ensure a low rate of false discovery (corrected to 1 in 10,000 SNPs). We then produced a Manhattan plot along with the positions of candidate SNPs. We identified the genomic coordinates of the candidate SNPs in the transcriptome-guided genome annotation to determine if they were genic or non-genic and the putative function of the gene or nearest candidate gene. If the gene was not annotated by FGENESH++, we queried the protein sequence against the National Center for Biotechnology Information (NCBI)’s RefSeq nonredundant protein sequences database using the BLASTp webserver for homology to known genes (Johnson et al. 2008).

## Results

### Genome sequencing and assembly

The Tasmanian scrubtit genome assembly was 1.48 Gb in length, comprising 1,516 contigs with a contig N50 of 7.715 Mb (Tables S3 and S4). Over 99.99% of raw reads were retained after quality trimming. The genome had 97.18% complete BUSCOs [Single copy: 96.4%; Duplicated: 0.7%], 0.5% fragmented BUSCOs, and 2.4% missing BUSCOs. A total of 12,877 predicted genes were used as evidence for genome annotation from the global transcriptome, which had 94.9% complete BUSCOs [Single copy: 15.0%; Duplicated: 79.9%], 1.3% fragmented BUSCOs, and 3.8% missing BUSCOs. After annotation, 30,347 genes were predicted, with an annotation completeness of 82.8% BUSCOs [Single copy: 82.1%; Duplicated: 0.7%], 7.7% fragmented BUSCOs, and 9.5% missing BUSCOs. The mitochondrial genome was 16,867 bp in length and was identified on a single contig (Fig. S5).

### Population genetic structure

**Fig. 2.**
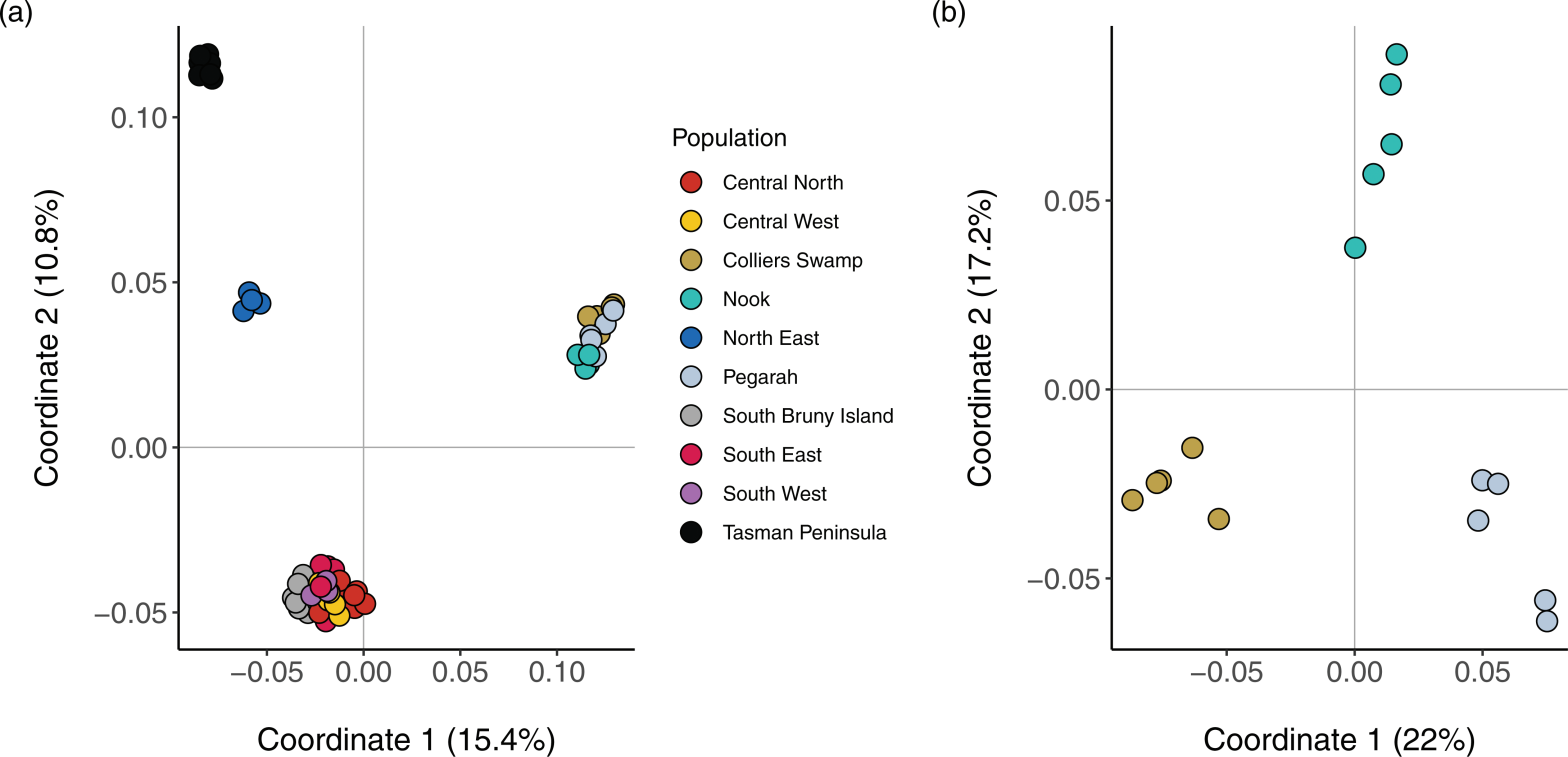
Multidimensional scaling plot (MDS) showing the genetic structure of scrubtit samples by population based on Prevosti’s genetic distance. a) shows all sampled individuals across mainland Tasmania and King Island; b) shows only King Island samples. Numbers in parentheses on axis titles denote the proportion of total variance explained by the first two coordinates.

The MDS plots showed evidence of four distinct genetic clusters in the scrubtit (Fig. 2a). Within mainland Tasmania, individuals sampled in the northeast of the state and on the Tasman Peninsula were distinct from the rest of the mainland population. King Island scrubtits formed their own cluster distinct from the Tasmanian scrubtit, with additional substructuring into three subpopulations when analyzed independently (Fig. 2b).

Pairwise *F*_ST_ estimates between the King Island subpopulations (0.12 to 0.18) were larger than the majority of the pairwise estimates between the mainland subpopulations (0.01 to 0.18) except the Tasman Peninsula (>0.22, Table S5). Genetic isolation was positively correlated with geographic distance (glm β = 494.7, SE = 101.7, *P* < 0.001, McFadden’s *R*^2^ = 0.36, Fig. S6).

**Fig. 3.**
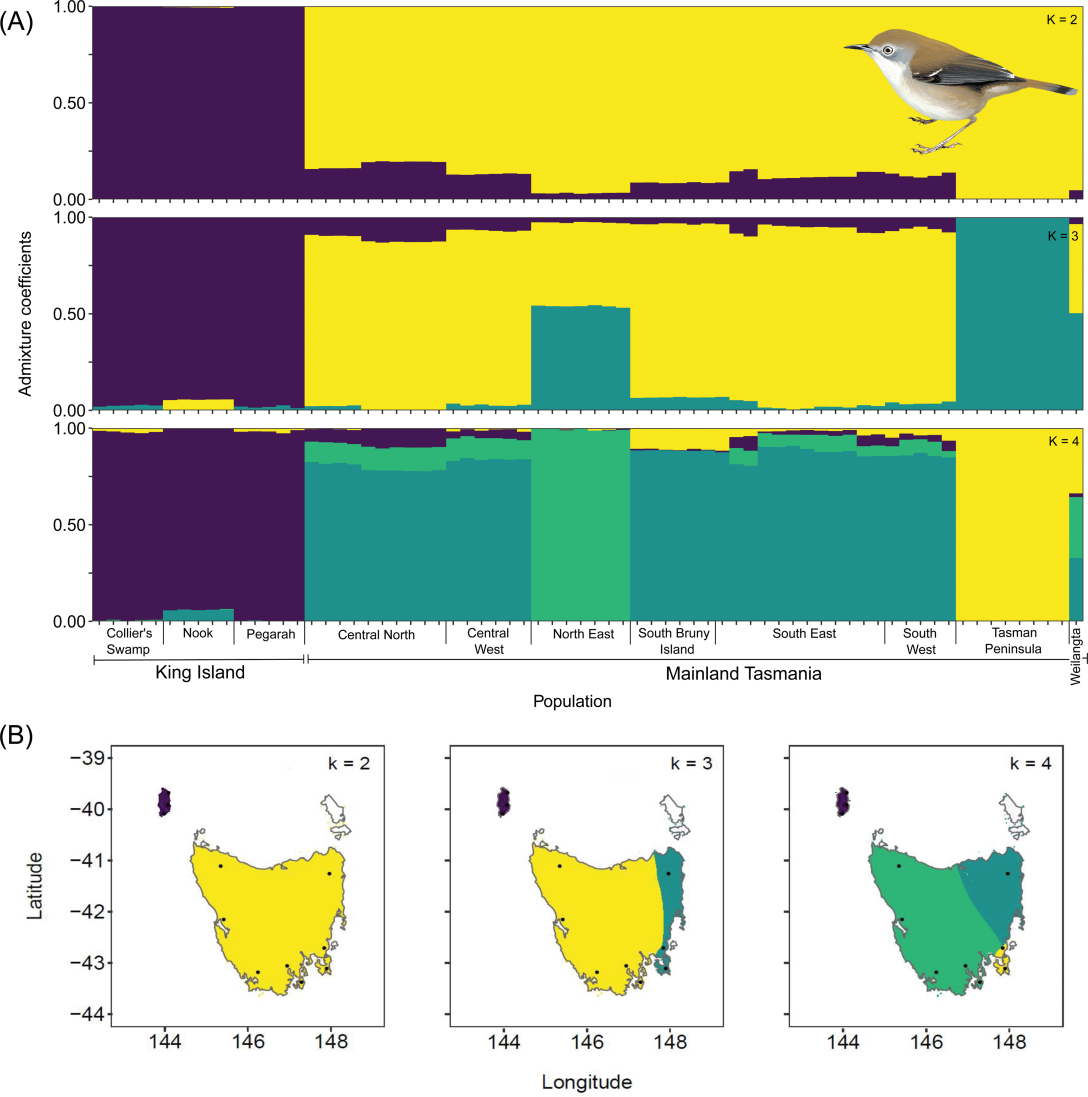
a) Admixture plots showing the probability of assignment of each individual scrubtit to a-priori subpopulations based on sampling locations when the number of ancestral populations (*k*) ranged from 2 to 4; b) Patterns of landscape genomic structure across the geographical range of the scrubtit. Each panel shows the population genomic structure when two, three, or four ancestral clusters (*k* values) are identified in the data. Colors in each panel represent the distribution of an ancestral cluster, interpolated across the distribution of the species. Black points indicate sampling locations.

**Fig. 4.**
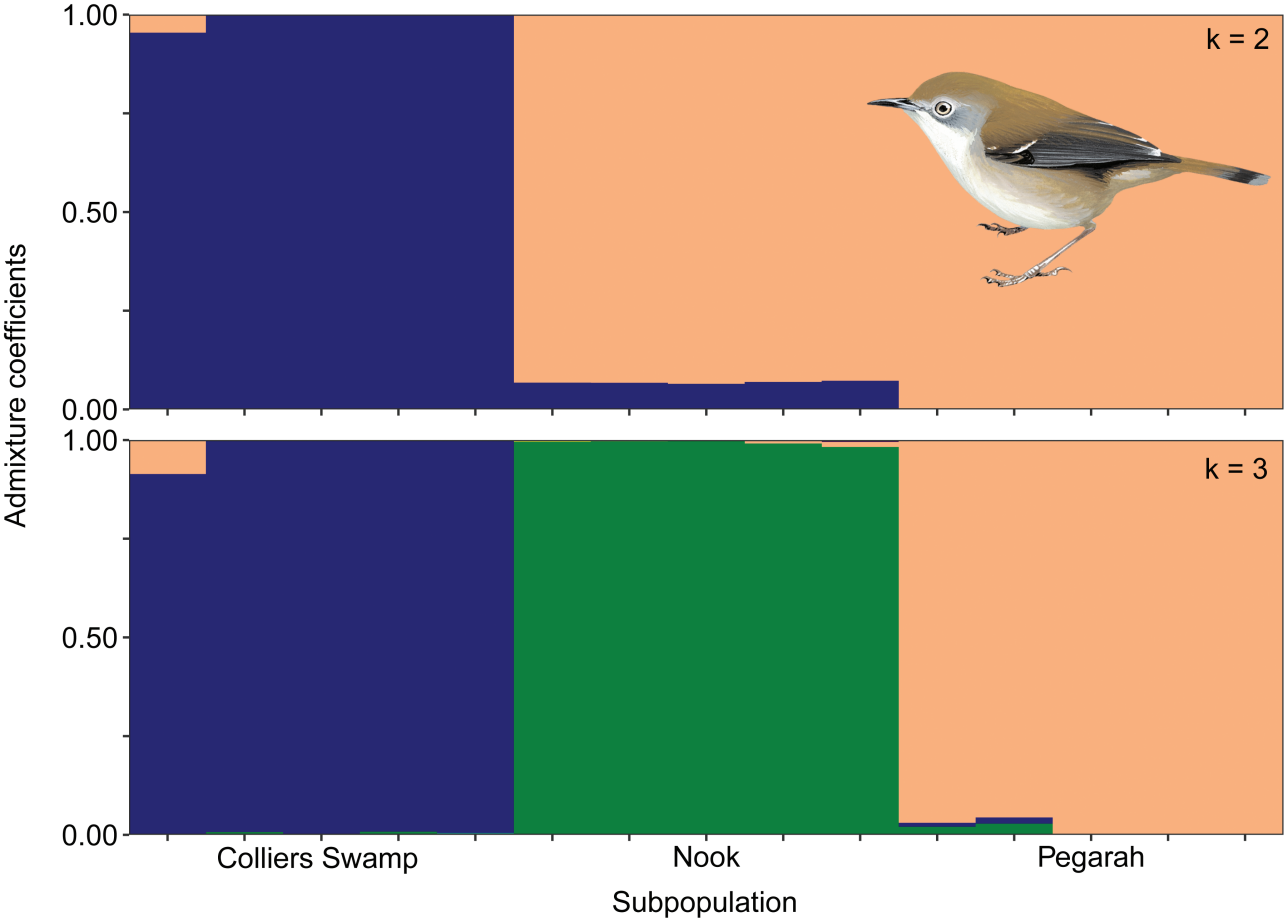
Admixture plots showing the probability of assignment of individual King Island scrubtits to a priori subpopulations based on sampling locations when the number of ancestral populations (*k*) ranged from 2 to 3.

Increasing values of *k* resulted in decreased values of the cross-entropy criterion (Fig. S7), with changes in the criterion value suggesting that the best number of ancestral populations for interpretation within the current sampling design ranged from 2 to 4. Admixture plots showed strong differentiation of the King Island subspecies from the Tasmanian scrubtit regardless of the estimated number of ancestral populations (Fig. 3). When *k* = 3, the east coast subpopulations in the Tasman Peninsula and north east separated with a high degree of confidence from the remainder of the Tasmanian scrubtit population, and when *k* = 4 the Tasman Peninsula and north east subpopulations separated from each other. Despite being isolated from the mainland by a 4 km wide sea strait, the Bruny Island subpopulation was less isolated from the rest of the Tasmanian scrubtit population than were the north-eastern and Tasman Peninsula subpopulations (Fig. 3, Table S5). When analyzed independently, King Island scrubtits showed a high degree of differentiation by subpopulation when *k *= 3 (Figs. 4, S9).

### Genetic diversity, inbreeding, and mean kinship

**Fig. 5.**
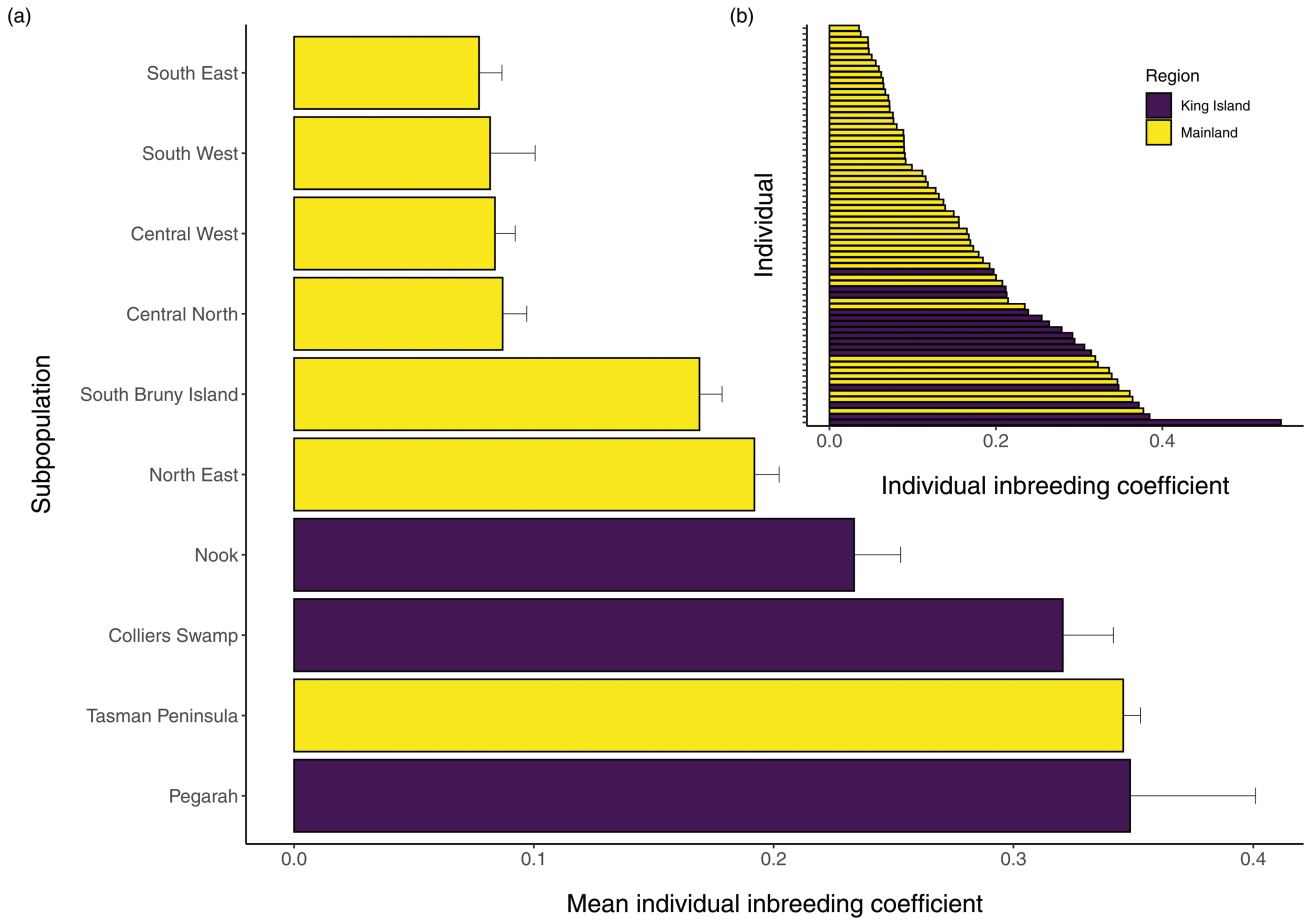
Inbreeding coefficients for King Island and Tasmanian scrubtits. Shown are a) averages (±standard error) across subpopulations and b) estimates for all individuals.

Measures of the mean number of alleles, effective number of alleles, expected and observed heterozygosity, and allelic richness were all lower in the three King Island scrubtit subpopulations than they were within all of the Tasmanian scrubtit subpopulations, except the Tasman Peninsula (Table 1, Fig. S10). Mean individual level inbreeding coefficients calculated using the modified Visscher’s method were higher in the three King Island scrubtit populations than all of the Tasmanian scrubtit populations, again except the Tasman Peninsula (Fig. 5a). The majority of King Island scrubtits plotted toward the higher end of individual inbreeding estimates across the entire sample (Fig. 5b). The mean number of private alleles within the King Island scrubtit subpopulations were within the range of those of the Tasmanian scrubtit, but the number increased 3- to 6-fold when King Island scrubtits were analyzed separately (Table 1). Within population mean kinships for the King Island populations when analyzed as the island populations only, reflected on average a first cousin relationship (~0.0625; Table 1). However, when within-population mean kinships for King Island populations were analyzed with all the Tasmanian mainland populations, the within-populations mean kinship for the King Island populations reflected on average a full-sibling relationship/parent-offspring (~0.2500; Table 1). Samples from the Tasman Peninsula also reflected a mean kinship of a full-sibling/parent–offspring relationship, whilst the North East and South Bruny Island samples reflect a mean kinship of a half-sibling relationship (Table 1).

### Genetic relationship with baldness

**Fig. 6.**
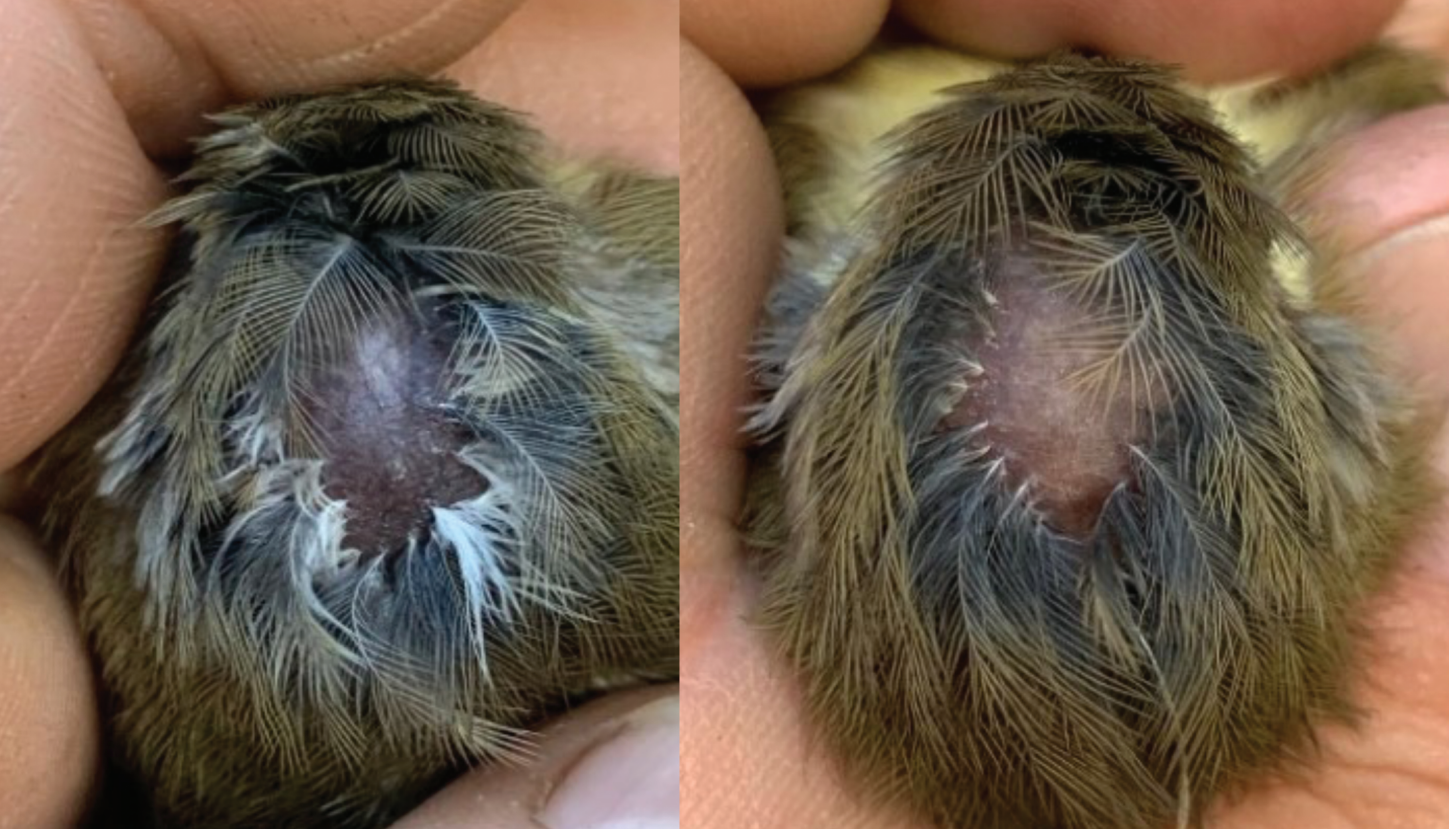
Examples of crown baldness in King Island scrubtits. See Fig. S3 for additional examples.

Crown baldness was present in 8/15 (53% of) King Island scrubtits (Fig. 6), including in both sexes and all three subpopulations, but none of the 55 Tasmanian scrubtits. Logistic regression across King Island scrubtits showed no relationship between multi-locus heterozygosity (MLH) and the probability of crown baldness occurrence (β = 11.56, SE = 21.8, *z* = 0.53, *P* = 0.60, Fig. S4). After accounting for population structure (*k* = 3) and adjusting the *P*-values, the LFMM identified six loci that were significantly associated with baldness (Fig. S11). Of the six candidate SNPs, three were genic and three were non-genic (Table S7). Three SNPs were located on the same assembled contig, including one in the *DOCK11* gene involved in regulation of filopodium assembly. Filopodia have been implicated in feather follicle formation and feather branching in chickens (Cheng et al. 2018), suggesting a possible role of this gene in baldness.

## Discussion

We explored the population genomic status of the critically endangered King Island scrubtit in the context of its mainland conspecific—the Tasmanian scrubtit—using a genome-wide SNP dataset developed with samples collected from across their contemporary distribution. We also provide the first sequenced and assembled long-read genome for the family *Acanthizidae.* We show that the scrubtit population is structured into four genetic clusters, with additional substructuring of the King Island scrubtit population when analyzed independently. Genomic diversity is lower and inbreeding coefficients are higher in the King Island scrubtit than in the Tasmanian scrubtit. Crown baldness was present in over 50% of King Island scrubtits, but none of the 55 Tasmanian scrubtits. Baldness was not linked to particularly low levels of multi-locus heterozygosity in King Island scrubtits, but was significantly associated with six candidate SNPs. Below, we discuss the implications of these results for research and genetic management requirements of the King Island scrubtit.

### Population genomic structure

The patterns of population genomic structure in scrubtits add further evidence that Tasmanian biodiversity is broadly structured by biogeographic barriers related to climate, topology, and anthropogenic impacts. Gene flow between King Island and the mainland probably ceased toward the end of the Pleistocene glacial period around 12,000 years ago, when sea-level rise flooded low-lying marshlands in what is now the Bass Strait (Bowdler 2015). King Island scrubtits are already restricted to a climate refuge, and habitat loss following European settlement has fragmented this refuge into three isolated subpopulations. Our results suggest these subpopulations are now unlikely to be connected by natural gene flow, with genetic differentiation between the subpopulations resulting from vicariance and genetic drift. Pairwise *F*_ST_ values between King Island subpopulations are similar to or greater than those between Tasmanian scrubtit subpopulations, despite the substantially smaller geographic distances between those on King Island (circa 20 km) than those on the Tasmanian mainland (60 to 100 km). We suggest that this allelic isolation is a product of two driving forces: 1) the highly modified matrix between King Island subpopulations preventing dispersal of individuals among localities (i.e. habitat fragmentation), and 2) the process of genetic drift occurring rapidly due to small population sizes.

Among Tasmanian scrubtits, east coast birds are isolated somewhat from the rest of the population by a broad swathe of unsuitable habitats associated with a warmer and drier climate in the midlands (Corney et al. 2013), comprising predominantly dry sclerophyll forest that has also been heavily cleared since European arrival (Fig. 1). The distribution of wet sclerophyll forest and rainforest within the east coast is patchy. Tasman Peninsula scrubtits are isolated by ocean on three sides and a narrow neck of land on the fourth, but dispersal is likely also limited by a lack of wet forest and land clearing on the nearby mainland (Fig. 1). This pattern of genetic isolation of the Tasman Peninsula scrubtit is similar to the patterns observed in other taxa including the Tasmanian devil *Sarcophilus harrisii* (Jones et al. 2004; Farquharson et al. 2022) and mountain ash *Eucalyptus regnans* (von Takach et al. 2021). In contrast, wet forest is abundant on the mainland adjacent to south Bruny Island. This suggests occasional gene flow across the 4 km strait separating Bruny Island from the Tasmanian mainland can occur, as has been demonstrated in endangered 40-spotted pardalotes *Pardalotus quadragintus* (Alves et al. 2023).

### Genetic diversity, inbreeding, and mean kinship

Consistent with our predictions, observed levels of genetic diversity were significantly lower in King Island scrubits than in all Tasmanian scrubtit subpopulations apart from the Tasman Peninsula. Interestingly, the samples from the Tasman Peninsula exhibit similar within-population mean kinship values to those on King Island. Relatively lower levels of genomic diversity are observed in many island vertebrate populations, particularly on small islands, as shown in northern quolls *Dasyurus hallucatus* (von Takach et al. 2022) and black-footed tree rats (Djintamoonga) *Mesembriomys gouldii* (von Takach et al. 2023). As predicted due to the small fragmented population on King Island, individual inbreeding coefficients and mean kinship measures were generally higher in the King Island scrubtit than in the Tasmanian scrubtit, although high levels of inbreeding and relatedness were also observed in the Tasman Peninsula population of Tasmanian scrubtits. Highlighting that the King Island populations are likely to succumb to small population pressures, all individuals sampled within the King Island populations showed a degree of genetic relatedness approximating that of outbred first cousins within their respective subpopulations (MK ~0.0625; Table 1). There was no relatedness between the three King Island subpopulations, that is, Collier’s Swamp to Nook, MK = 0.000; Collier’s Swamp to Peragah, MK = 0.000; and Nook to Peragah, MK = 0.000, indicating there is likely to be little movement and breeding between these subpopulations.

Phenotypic evidence of defective traits in small, inbred populations is likely to occur but not always noticed in wild systems (but see e.g. Roelke et al. 1993 and Harrisson et al. 2019). Although unknown at this time if it is a defective trait or not, over 50% of King Island scrubtits we sampled exhibited crown baldness (Figs. 5 and S3). Baldness was present in both sexes and all three King Island subpopulations, but was not recorded in any Tasmanian scrubtits. The probability of baldness was negatively associated with multi-locus heterozygosity, but the relationship disappeared when we restricted the analysis to the King Island population. It is possible that baldness may be due to nongenetic effects such as endemic parasitism, disease, inter/intraspecific aggression linked to low habitat availability or an aging population (Lachish et al. 2012; Thys et al. 2017; van Velden et al. 2017). Unfortunately, we were unable to estimate accurately the age of the birds sampled. Baldness was similar in all affected individuals (Fig. S3) and we found six candidate SNPs that may play a role in the development or expression of this trait. One of these SNPs is associated with the DOCK11 gene, a gene that has been linked to early feather development in chickens (Fig. S10; Table S7; Cheng et al. 2018). Such genome-wide association studies can be limited using ddRAD data (Lowry et al. 2017), so further investigation into the phenotype-genotype associations is required to determine the genetic and physiological pathways leading to baldness.

### Conservation implications

The principal threat to persistence of the King Island scrubtit is habitat loss and its small population size (Threatened Species Section 2012). Preservation of remaining habitat and restoration of lost habitat will therefore be critical if the taxon is to recover in the longer term (Webb et al. 2016). In the shorter term, our results suggest the Pegarah Forest, Colliers Swamp and Nook Swamps subpopulations of the King Island scrubtit should be managed as a single management unit with translocations between these small, isolated populations used to improve gene flow that has been lost due to habitat fragmentation and ensure that the current level of overall genetic diversity is maintained (Frankham et al. 2017). Higher levels of inbreeding and relatedness, and higher probability of visible defective traits (i.e. baldness) relative to the Tasmanian scrubtit suggest that genetic factors may well be exacerbating population decline in King Island scrubtits. Future genetic management of the population is therefore warranted. There are currently no demographic data on dispersal and breeding success rates in King Island scrubtits. However, if breeding success is low and/ or contemporary gene flow between subpopulations is as infrequent as our results suggest, genetic rescue through the translocation of scrubtits between King Island subpopulations could help (Harrisson et al. 2016).

Action paralysis is a conscious management decision when threats are known, and risks of adverse events may be worth taking if a species or population is already known to be on a course for imminent extinction (Webb et al. 2018; Canessa et al. 2020). For many years, the risk of outbreeding depression has been used as an excuse for inaction (Avise and Nelson 1989; Ralls et al. 2018), but evidence for outbreeding depression is limited and predictable (Frankham et al. 2011, Frankham 2015). While we did not find any adaptive loci based on two relatively simple outlier tests, any future analysis into the risk of outbreeding depression could include research into the genomic architecture of adaptation in the species. A very real risk of any translocation is that introduced individuals may be vectors for the establishment of novel pathogens in threatened populations (Peters et al. 2014), but this risk can be mitigated with disease screening.

The current rate of King Island scrubtit population decline, and the extent to which this decline is exacerbated by genetic effects are currently unknown. Our results suggest two precautionary approaches to genetic management of King Island scrubtits are feasible. Assuming that delaying management will not compromise the chances of future King Island scrubtit population recovery, translocation of individuals either within the Tasman Peninsula subpopulation of Tasmanian scrubtits or from elsewhere on mainland Tasmania could be used as a trial for genetic rescue, given the remarkable similarity of this subpopulation’s genetic parameters to the King Island scrubtit. If conservation actions are considered urgent, genetic rescue trials could occur in the Pegarah Forest King Island scrubtit subpopulation, which our data suggest is most at risk of inbreeding and extinction, with minimal risks of negative genetic effects spreading to the other two subpopulations via natural gene flow.

### Research implications

Field research to understand current vital rates and dispersal dynamics is urgently required to quantify the fitness costs of high inbreeding and low genetic diversity in the King Island scrubtit as these costs can be high (Harrisson et al. 2019; Kardos et al. 2023). Accurate estimation of the King Island scrubtit effective population size (Ne) is also a research priority. We attempted to estimate Ne but the derived estimates were implausible, likely due to violations of key assumptions and our relatively small sample sizes (Do et al. 2014). With these data, the relative contribution of genetic effects associated with the taxon’s small and fragmented population to its decline can then be compared to other known threats such as predation by feral cats. This information can help prioritize implementation of recovery actions to address the most prominent threats facing the taxon. We consider two particular priorities are to determine: 1) whether contemporary breeding success and juvenile recruitment in King Island scrubtit are low, and if so whether this is primarily due to inbreeding depression (e.g. Duntsch et al. 2023), high predation rates (e.g. Crates et al. 2019) or simply a severe shortage of breeding resources (i.e. habitat saturation, Komdeur 1992); and 2) whether any surviving juveniles are able to successfully disperse between subpopulations. Our data suggest successful juvenile dispersal is unlikely, in which case juveniles 1) remain in their natal areas without breeding; 2) breed with close relatives; or 3) die during dispersal. Under such scenarios, translocation of juveniles between subpopulations could facilitate genetic rescue with minimal risk to the current effective population (Frankham et al. 2015).

More broadly, our study highlights the potential for avoidable biodiversity loss to occur when the conservation requirements of less enigmatic or geographically remote taxa are overlooked (Woinarski et al. 2017). It also highlights the challenges of implementing effective conservation measures when basic population monitoring data are lacking (Lindenmayer et al. 2020). The conservation status of the King Island scrubtit has been known for decades (Garnett et al. 2011), but targeted research to establish the species’ basic ecological requirements is ongoing (Webb et al. 2016; Bell et al. 2023). Population genomics is itself an important tool for implementing evidence-based conservation, but is most effective at preventing extinctions when complemented with rigorous, field-based population monitoring data (Taylor et al. 2017, Duntsch et al. 2023). With sufficient funding, evidence-based actions can then ensure no more preventable extinctions occur (Sutherland et al. 2004).

## Supplementary material

Supplementary material is available at *Journal of Heredity* online.

esae029_suppl_Supplementary_Materials

## Data Availability

Related metadata and code, including georeferences in decimal degrees and date/month/year of sampling event and unique sample identifier tags that can be matched to the deposited genetic data, is available in the supplementary material and via the Dryad digital repository (Crates 2024) https://doi.org/10.5061/dryad.12jm63z66. The genome assembly and raw transcriptome data are made available under the NCBI BioProject PRJNA1014961. The raw PacBio HiFi reads are publicly available from the Bioplatforms Australia Threatened Species Initiative: https://data.bioplatforms.com/organization/threatened species. The assembled genome, global transcriptome, and genome annotation generated in this study are available on Amazon Web Services Australasian Genomes Open Data Store: https://awgg-lab.github.io/australasiangenomes/genomes.html.
