# Supplementary material for: Genomic insights into the critically endangered King Island scrubtit
Source: J Hered. 2024 May 30;115(5):552–64. doi: 10.1093/jhered/esae029 (PMC11334212; doi:10.1093/jhered/esae029)
Supplement: esae029_suppl_Supplementary_Materials [file esae029_suppl_supplementary_materials.docx]

**SUPPLEMENTARY INFORMATION**

**Supplementary file S1: Additional information on methods**

*Molecular sexing*

We determined the sex of individuals using a polymerase chain reaction (PCR) protocol adapted from Fridolfsson and Ellegren (1999). We used 0.25μL forward primer *2550F,* 0.25μL reverse primer *2718R* and 6.25μL One*Taq®* DNA Polymerase (*New England BioLabs*, Victoria, Australia) in combination with 1μL DNA for each of the 12.5μL reactions. We used an *Eppendorf® Mastercycler* machine with an annealing temperature of 48°C. To visualise the reactions, we ran the PCRs at 100V for 30 minutes on a 1.5% agarose gel with *sybr* stain (*Invitrogen*, NSW, Australia). Males show one band and females show two.

*Library preparation*

The library preparation protocol consisted of (i) digestion using the aforementioned restriction enzymes (ii) ligation with one of 48 unique inline barcoded adapters compatible with the restriction site overhang, (iii) manual sample pooling, (iv) DNA purification (QIAquick PCR Purification Kit followed by SPRIselect paramagnetic beads), (v) 62 bp narrow size-selection targeting fragments of 280– 342 bp in length (BluePippin, Sage Science) and (vi) a PCR (polymerase chain reaction) amplification step where one of two multiplexing index primers was added. Indexed libraries were pooled together and loaded onto flow cells for 150- bp paired-end sequencing on an Illumina NovaSeq 6000 platform.

*Bioinformatics pipeline*

Raw sequence data were demultiplexed using the *process_radtags* function of Stacks (Catchen et al., 2013), with checking for intact RAD sites and reads quality-checked and trimmed. Of the 503.8 million total reads, 99.9% were retained after demultiplexing. Trimmomatic v0.39 (Bolger et al., 2014) was used to remove possible adapter contamination, and reads were aligned to the reference genome assembled in this study with BWA v0.7.17-r1188 (Li & Durbin, 2009). All samples mapped well (mean mapping rate = 95.16%; SD = 0.67, range = 92.83–96.46%). The *gstacks* function of Stacks was used to call a catalogue of variants, filtered with the *populations* function by retaining variants in with a minor allele frequency of 0.01; maximum heterozygosity of 0.8; genotyped in 30% of samples; and retaining only one SNP per tag (--write-random-snp). SNPs were filtered using a custom R script to retain SNPs with a minimum average allelic depth of 2.5× per allele; a coverage difference between alleles of ≤ 80%; a genotyping rate per locus ≥ 80%; and a reproducibility of genotype calls between replicates of 100% (Wright et al., 2019). The mean error rate between replicate pairs was 1.17% (SD = 5.58; range = 0.60–2.19%). Further filtering of the SNPs is outlined in Table S2.

**Supplementary file S2: Genome sequencing and assembly methodology**

*Sample collection and DNA/RNA extraction*

A single adult male Tasmanian scrubtit was captured using a mist net at Weilangta Forest in south-eastern Tasmania and transported immediately to Hobart where it was euthanized by a veterinary surgeon (Australian National University Animal Research Authority A2021/33). The sample was dissected at the Tasmanian Museum and Art Gallery specimens collection, where brain and organ tissue were preserved in RNALater before being stored at -80˚C. High molecular weight (HMW) DNA was extracted from heart and kidney tissue using the Nanobind Tissue Big DNA Kit v1.0 11/19 (Circulomics). A Qubit fluorometer was used to assess the concentration of DNA with the Qubit dsDNA BR assay kit (Thermo Fisher Scientific). RNA was extracted from six organs preserved in RNALater, using the RNeasy Plus Mini Kit (Qiagen) with RNAse-free DNAse (Qiagen) digestion. Extractions were performed using tissue from the heart, brain, spleen, kidney, liver and gonads.

*Library construction and sequencing*

HMW DNA was sent for PacBio HiFi library preparation with Pippin Prep and sequencing on one single molecule real-time (SMRT) cell of the PacBio Sequel II (Australian Genome Research Facility, Brisbane, Australia).

Total RNA was sequenced as 100 bp paired-end reads using Illumina NovaSeq 6000 with Illumina Stranded mRNA library preparation at the Ramaciotti Centre for Genomics (University of New South Wales, Sydney, Australia).

*Genome assembly*

Genome assembly was conducted on Galaxy Australia (The Galaxy Community, 2022) following the genome assembly guide (Price & Farquharson, 2022). HiFiAdapterFilt was used to remove adapter sequence in the raw PacBio HiFi reads (Sim et al., 2022), followed by assembly with HiFiasm v0.16.1 with default parameters (Cheng et al., 2021; Cheng et al., 2022).

The quality of the genome assembly was assessed on Galaxy Australia with the genome assessment post-assembly workflow (Price, 2023). Basic genome assembly statistics were calculated with QUAST v5.0.2 (Mikheenko et al., 2018). Completeness was assessed using Benchmarking Universal Single-Copy Orthologs (BUSCO) v5.2.2 (Simão et al., 2015) with the Aves_odb10 lineage (n = 8,338 BUSCOs). The repetitive elements of the genome were identified and classified by building a custom database using RepeatModeler v2.0.1 (Flynn et al., 2020) and RepeatMasker v4.0.9 (Smit et al., 2013-2015) with the -nolow parameter to avoid masking of simple low-complexity repeats, run on a Pawsey Supercomputing Centre Nimbus cloud machine (256 GB RAM, 64 vCPU, 3 TB storage).

*Mitogenome assembly*

The mitochondrial genome was assembled from the primary genome assembly using MitoHiFi v3 (Uliano-Silva et al., 2022). MitoHiFi identified the rock warbler *Origma solitaria* (NCBI reference sequence NC_053100.1; Feng et al., 2020) as the most closely-related publicly available mitochondrial genome. The mitochondrial genome was visualised with MitoZ v2.3 (Meng et al., 2019).

*Transcriptome assembly*

Transcriptome assembly was conducted on the University of Sydney High Performance Computer, Artemis. FastQC v0.11.8 (Andrews, 2010) was used to assess the quality of raw reads. Trimmomatic v0.39 (Bolger et al., 2014) was used to quality trim reads specifying TruSeq3-PE adapters, SLIDINGWINDOW:4:5, LEADING:5, TRAILING:5 and MINLEN:25. The repeat-masked genome was indexed and reads aligned with HiSat2 v2.1.0 (Kim et al., 2019). SamTools v1.9 view and sort converted the files to coordinate-sorted BAM format. A GTF for each transcriptome was generated with StringTie v2.1.6 (Pertea et al., 2015). Aligned RNAseq reads were merged into transcripts and filtered to remove transcripts found in only one tissue with fragments per kilobase of transcript per million mapped fragments (FPKM) < 0.1, using TAMA-merge v2020/12/17 (Kuo et al., 2020) and CPC2 v2019-11-19 (Kang et al., 2017). TransDecoder v2.0.1 (Haas, 2022) predicted open reading frames in the resulting global transcriptome. The completeness of the global transcriptome was assessed using BUSCO v5.2.2 in ‘transcriptome’ mode with the Aves_odb10 lineage on Galaxy Australia.

*Genome annotation*

Genome annotation was performed using FGENESH++ v7.2.2 (Softberry; (Solovyev et al., 2006)) on a Pawsey Supercomputing Centre Nimbus cloud machine (256 GB RAM, 64 vCPU, 3 TB storage) using the longest open reading frame predicted from the global transcriptome, non-mammalian settings, and optimised parameters supplied with the *Corvus brachyrhynchos* (American crow) gene-finding matrix. BUSCO v5.2.2 in ‘protein’ mode was used to assess the completeness of the annotation with the Aves_odb10 lineage on Galaxy Australia.

**Supplementary References***

Allio, R., Schomaker-Bastos, A., Romiguier, J., Prosdocimi, F., Nabholz, B., & Delsuc, F. (2020). MitoFinder: Efficient automated large-scale extraction of mitogenomic data in target enrichment phylogenomics. *Molecular Ecology Resources*, *20*(4), 892-905. <https://doi.org/https://doi.org/10.1111/1755-0998.13160>

Andrews, S. (2010). *FastQC: A quality control tool for high throughput sequence data*. <http://www.bioinformatics.babraham.ac.uk/projects/fastqc/>

Barnett, D. W., Garrison, E. K., Quinlan, A. R., Strömberg, M. P., & Marth, G. T. (2011). BamTools: a C++ API and toolkit for analyzing and managing BAM files. *Bioinformatics*, *27*(12), 1691-1692. <https://doi.org/10.1093/bioinformatics/btr174>.

Bell, P. J., Webb, M. H., Holdsworth, M., & Baker, G. B. (2023). Defining and mapping habitat. requirements to support the survival of King Island threatened birds. Report to Cradle Coast NRM, Cradle Coast Authority, Burnie, Tasmania.

Bolger, A. M., Lohse, M., & Usadel, B. (2014). Trimmomatic: a flexible trimmer for Illumina sequence data. *Bioinformatics*, *30*(15), 2114-2120. <https://doi.org/10.1093/bioinformatics/btu170>

Bushnell, B. (2022). *BBMap*. Retrieved February 2022 from sourceforge.net/projects/bbmap/

Cheng, H., Concepcion, G. T., Feng, X., Zhang, H., & Li, H. (2021). Haplotype-resolved de novo assembly using phased assembly graphs with hifiasm. *Nature Methods*, *18*(2), 170-175. <https://doi.org/10.1038/s41592-020-01056-5>

Cheng, H., Jarvis, E. D., Fedrigo, O., Koepfli, K.-P., Urban, L., Gemmell, N. J., & Li, H. (2022). Haplotype-resolved assembly of diploid genomes without parental data. *Nature Biotechnology*, *40*(9), 1332-1335. <https://doi.org/10.1038/s41587-022-01261-x>

Danecek, P., Bonfield, J. K., Liddle, J., Marshall, J., Ohan, V., Pollard, M. O., Whitwham, A., Keane, T., McCarthy, S. A., Davies, R. M., & Li, H. (2021). Twelve years of SAMtools and BCFtools. *GigaScience*, *10*(2). <https://doi.org/10.1093/gigascience/giab008>

Eiler, A., Löfgren, A., Hjerne, O., Nordén, S., & Saetra, P. (2018). Environmental DNA (eDNA) detects the pool frog (*Pelophylax lessonae*) at times when traditional monitoring methods are insensitive. *SCIENTIFIC REPORTS*, *8*, 5452. <https://doi.org/10.1038/s41598-018-23740-5>

Feng, S., Stiller, J., Deng, Y. *et al.* Dense sampling of bird diversity increases power of comparative genomics. *Nature* **587**, 252–257 (2020). https://doi.org/10.1038/s41586-020-2873-9

Flynn, J. M., Hubley, R., Goubert, C., Rosen, J., Clark, A. G., Feschotte, C., & Smit, A. F. (2020). RepeatModeler2 for automated genomic discovery of transposable element families. *Proc Natl Acad Sci U S A*, *117*(17), 9451-9457. <https://doi.org/10.1073/pnas.1921046117>

Haas, B. J. (2022). *TransDecoder (find coding regions within transcripts)*. Retrieved February 2022 from <https://github.com/TransDecoder/TransDecoder>

IUCN SSC Amphibian Specialist Group. (2022). *Taudactylus pleione. The IUCN Red List of Threatened Species 2022:e.T21533A78446285.* Retrieved 31st March from <https://dx.doi.org/10.2305/IUCN.UK.2022-2.RLTS.T21533A78446285.en>

Kang, Y.-J., Yang, D.-C., Kong, L., Hou, M., Meng, Y.-Q., Wei, L., & Gao, G. (2017). CPC2: a fast and accurate coding potential calculator based on sequence intrinsic features. *Nucleic Acids Research*, *45*(W1), W12-W16. <https://doi.org/10.1093/nar/gkx428>

Kim, D., Paggi, J. M., Park, C., Bennett, C., & Salzberg, S. L. (2019). Graph-based genome alignment and genotyping with HISAT2 and HISAT-genotype. *Nature Biotechnology*, *37*(8), 907-915. <https://doi.org/10.1038/s41587-019-0201-4>

Kuo, R. I., Cheng, Y., Zhang, R., Brown, J. W. S., Smith, J., Archibald, A. L., & Burt, D. W. (2020). Illuminating the dark side of the human transcriptome with long read transcript sequencing. *BMC Genomics*, *21*(1), 751. <https://doi.org/10.1186/s12864-020-07123-7>

Li H. and Durbin R. (2009) Fast and accurate short read alignment with Burrows-Wheeler transform. *Bioinformatics*, **25**, 1754-1760.

Meng, G., Li, Y., Yang, C., & Liu, S. (2019). MitoZ: a toolkit for animal mitochondrial genome assembly, annotation and visualization. *Nucleic Acids Research*, *47*(11), e63-e63. <https://doi.org/10.1093/nar/gkz173>

Mikheenko,A., Prjibelski, A., Saveliev, V., Antipov, D., Gurevich, A., Versatile genome assembly evaluation with QUAST-LG, Bioinformatics (2018) 34 (13): i142-i150. doi: 10.1093/bioinformatics/bty266 First published online: June 27, 2018

Pertea, M., Pertea, G. M., Antonescu, C. M., Chang, T. C., Mendell, J. T., & Salzberg, S. L. (2015). StringTie enables improved reconstruction of a transcriptome from RNA-seq reads. *Nat Biotechnol*, *33*(3), 290-295. <https://doi.org/10.1038/nbt.3122>

Price, G., & Farquharson, K. (2022). PacBio HiFi genome assembly using hifiasm v2.1. WorkflowHub. <https://doi.org/10.48546/WORKFLOWHUB.WORKFLOW.221.3>

Price, G. (2023). Genome assessment post assembly. WorkflowHub. <https://doi.org/10.48546/WORKFLOWHUB.WORKFLOW.403.2>

Simão, F. A., Waterhouse, R. M., Ioannidis, P., Kriventseva, E. V., & Zdobnov, E. M. (2015). BUSCO: assessing genome assembly and annotation completeness with single-copy orthologs. *Bioinformatics*, *31*(19), 3210-3212. <https://doi.org/10.1093/bioinformatics/btv351>

Smit, A. F. A., Hubley, R., & Green, P. (2013-2015). *RepeatMasker Open-4.0*. Retrieved February 2022 from <http://www.repeatmasker.org>

Solovyev, V., Kosarev, P., Seledsov, I., & Vorobyev, D. (2006). Automatic annotation of eukaryotic genes, pseudogenes and promoters. *Genome Biology*, *7*(1), S10. <https://doi.org/10.1186/gb-2006-7-s1-s10>

Sim, S.B., Corpuz, R.L., Simmonds, T.J. *et al.* HiFiAdapterFilt, a memory efficient read processing pipeline, prevents occurrence of adapter sequence in PacBio HiFi reads and their negative impacts on genome assembly. *BMC Genomics* **23**, 157 (2022). https://doi.org/10.1186/s12864-022-08375-1

Sun, Y. B., Zhang, Y., & Wang, K. (2020). Perspectives on studying molecular adaptations of amphibians in the genomic era. *Zool Res*, *41*(4), 351-364. <https://doi.org/10.24272/j.issn.2095-8137.2020.046>

The Galaxy Community. (2022). The Galaxy platform for accessible, reproducible and collaborative biomedical analyses: 2022 update. *Nucleic Acids Research*, *50*(W1), W345-W351. <https://doi.org/10.1093/nar/gkac247>

Uliano-Silva, M., Gabriel R. N. Ferreira, J., Krasheninnikova, K., Formenti, G., Abueg, L., Torrance, J., Myers, E. W., Durbin, R., Blaxter, M., & McCarthy, S. A. (2023). MitoHiFi: a python pipeline for mitochondrial genome assembly from PacBio High Fidelity reads. *bioRxiv*, 2022.2012.2023.521667. <https://doi.org/10.1101/2022.12.23.521667>

Vurture, G. W., Sedlazeck, F. J., Nattestad, M., Underwood, C. J., Fang, H., Gurtowski, J., & Schatz, M. C. (2017). GenomeScope: fast reference-free genome profiling from short reads. *Bioinformatics*, *33*(14), 2202-2204. <https://doi.org/10.1093/bioinformatics/btx153>

Webb, M. H., Holdsworth, M., Stojanovic, D., Terauds, A., Bell, P., & Heinsohn, R. (2016). Immediate action required to prevent another Australian avian extinction: the King Island Scrubtit. Emu-Austral Ornithology, 116(3), 223-229.

Wright, B., Farquharson, K.A., McLennan, E.A., Belov, K., Hogg, C.J., Grueber, C.E. (2019). From reference genomes to population genomics: comparing three reference-aligned reduced-representation sequencing pipelines in two wildlife species. BMC Genomics. 20(453). doi:10.1186/s12864-019-5806-y

**Supplementary Tables**

**Table S1:** King Island and Tasmanian scrubtit sample metadata.

| Source | Sample | Specimen | Tissue | Pres. | Taxon | Scientific name | Collection | Latitude | Longitude | genotypic sex | Health | DNA conc |
| --- | --- | --- | --- | --- | --- | --- | --- | --- | --- | --- | --- | --- |
| population | id | id | type | Temp. | id |  | date |  |  |  | state | Ng/ul |
| Weilangta | 407004 | ANU-ST67 | Muscle in EtOh | -80 | 720576 | *Acanthornis magna magna* | 14/04/2022 | -42.7095 | 147.8403 | male | collected for genome sequencing | 284 |
| Weilangta | 407008 | ANU-ST67 | Muscle in EtOh | -80 | 720576 | *Acanthornis magna magna* | 14/04/2022 | -42.7095 | 147.8403 | male | collected for genome sequencing | 284 |
| Colliers Swamp | 406973 | ANU-ST36 | Blood in EtOh | -80 | 720576 | *Acanthornis magna greeniana* | 7/04/2022 | -40.09526 | 143.96423 | male | released live and healthy | 18.9 |
| Colliers Swamp | 406974 | ANU-ST37 | Blood in EtOh | -80 | 720576 | *Acanthornis magna greeniana* | 7/04/2022 | -40.089798 | 143.9824 | male | released live and healthy | 15.9 |
| Colliers Swamp | 406975 | ANU-ST38 | Blood in EtOh | -80 | 720576 | *Acanthornis magna greeniana* | 7/04/2022 | -40.089358 | 143.9793 | male | released live and healthy. Bald head | 26.8 |
| Colliers Swamp | 406976 | ANU-ST39 | Blood in EtOh | -80 | 720576 | *Acanthornis magna greeniana* | 7/04/2022 | -40.089358 | 143.9793 | female | released live and healthy | 15.8 |
| Colliers Swamp | 406977 | ANU-ST40 | Blood in EtOh | -80 | 720576 | *Acanthornis magna greeniana* | 7/04/2022 | -40.0876 | 143.9846 | male | released live and healthy. Bald head | 72.8 |
| Nook | 406978 | ANU-ST41 | Blood in EtOh | -80 | 720576 | *Acanthornis magna greeniana* | 9/04/2022 | -39.6735 | 144.0884 | female | released live and healthy | 38.1 |
| Nook | 406979 | ANU-ST42 | Blood in EtOh | -80 | 720576 | *Acanthornis magna greeniana* | 9/04/2022 | -39.6735 | 144.0884 | male | released live and healthy. Bald head | 35.6 |
| Nook | 406980 | ANU-ST43 | Blood in EtOh | -80 | 720576 | *Acanthornis magna greeniana* | 9/04/2022 | -39.674752 | 144.0871 | female | released live and healthy. Bald head | 171 |
| Nook | 406981 | ANU-ST44 | Blood in EtOh | -80 | 720576 | *Acanthornis magna greeniana* | 9/04/2022 | -39.674752 | 144.0871 | male | released live and healthy | 78.7 |
| Nook | 406982 | ANU-ST45 | Blood in EtOh | -80 | 720576 | *Acanthornis magna greeniana* | 9/04/2022 | -39.674752 | 144.0871 | male | released live and healthy. Bald head | 31.6 |
| Nook | 407012 | ANU-ST43 | Blood in EtOh | -80 | 720576 | *Acanthornis magna greeniana* | 9/04/2022 | -39.674752 | 144.0871 | female | released live and healthy. Bald head | 171 |
| Pegarah | 406983 | ANU-ST46 | Blood in EtOh | -80 | 720576 | *Acanthornis magna greeniana* | 10/04/2022 | -39.92019 | 144.0597 | female | released live and healthy. Bald head | 110 |
| Pegarah | 406984 | ANU-ST47 | Blood in EtOh | -80 | 720576 | *Acanthornis magna greeniana* | 10/04/2022 | -39.92019 | 144.0597 | male | released live and healthy. Bald head | 157 |
| Pegarah | 406985 | ANU-ST48 | Blood in EtOh | -80 | 720576 | *Acanthornis magna greeniana* | 10/04/2022 | -39.907 | 144.094 | female | released live and healthy | 110 |
| Pegarah | 406986 | ANU-ST49 | Blood in EtOh | -80 | 720576 | *Acanthornis magna greeniana* | 10/04/2022 | -39.907 | 144.094 | male | released live and healthy | 118 |
| Pegarah | 406987 | ANU-ST50 | Blood in EtOh | -80 | 720576 | *Acanthornis magna greeniana* | 10/04/2022 | -39.901 | 144.088 | male | released live and healthy. Bald head | 67.9 |
| Central North | 406988 | ANU-ST51 | Blood in EtOh | -80 | 720576 | *Acanthornis magna magna* | 12/04/2022 | -41.0816 | 145.9205 | male | released live and healthy | 139 |
| Central North | 406989 | ANU-ST52 | Blood in EtOh | -80 | 720576 | *Acanthornis magna magna* | 12/04/2022 | -41.0816 | 145.9205 | female | released live and healthy. | 99 |
| Central North | 406990 | ANU-ST53 | Blood in EtOh | -80 | 720576 | *Acanthornis magna magna* | 12/04/2022 | -41.0816 | 145.9205 | male | released live and healthy | 30 |
| Central North | 406991 | ANU-ST54 | Blood in EtOh | -80 | 720576 | *Acanthornis magna magna* | 12/04/2022 | -41.0851 | 145.9218 | male | released live and healthy | 83.5 |
| Central North | 406992 | ANU-ST55 | Blood in EtOh | -80 | 720576 | *Acanthornis magna magna* | 12/04/2022 | -41.1273 | 144.9611 | male | released live and healthy | 108 |
| Central North | 406993 | ANU-ST56 | Blood in EtOh | -80 | 720576 | *Acanthornis magna magna* | 12/04/2022 | -41.1259 | 144.9603 | male | released live and healthy | 30 |
| Central North | 406994 | ANU-ST57 | Blood in EtOh | -80 | 720576 | *Acanthornis magna magna* | 12/04/2022 | -41.1259 | 144.9603 | female | released live and healthy | 35.3 |
| Central North | 406995 | ANU-ST58 | Blood in EtOh | -80 | 720576 | *Acanthornis magna magna* | 12/04/2022 | -41.1259 | 144.9603 | male | released live and healthy | 71.2 |
| Central North | 406996 | ANU-ST59 | Blood in EtOh | -80 | 720576 | *Acanthornis magna magna* | 12/04/2022 | -41.1259 | 144.9603 | female | released live and healthy | 42.2 |
| Central North | 406997 | ANU-ST60 | Blood in EtOh | -80 | 720576 | *Acanthornis magna magna* | 12/04/2022 | -41.1259 | 144.9603 | male | released live and healthy | 44 |
| Central West | 406998 | ANU-ST61 | Blood in EtOh | -80 | 720576 | *Acanthornis magna magna* | 13/04/2022 | -42.1525 | 145.3377 | female | released live and healthy | 163 |
| Central West | 406999 | ANU-ST62 | Blood in EtOh | -80 | 720576 | *Acanthornis magna magna* | 13/04/2022 | -42.1506 | 145.3434 | male | released live and healthy | 96.1 |
| Central West | 407000 | ANU-ST63 | Blood in EtOh | -80 | 720576 | *Acanthornis magna magna* | 13/04/2022 | -42.1568 | 145.446 | male | released live and healthy | 124 |
| Central West | 407001 | ANU-ST64 | Blood in EtOh | -80 | 720576 | *Acanthornis magna magna* | 13/04/2022 | -42.1568 | 145.446 | female | released live and healthy | 113 |
| Central West | 407002 | ANU-ST65 | Blood in EtOh | -80 | 720576 | *Acanthornis magna magna* | 13/04/2022 | -42.1466 | 145.4661 | male | released live and healthy | 106 |
| Central West | 407003 | ANU-ST66 | Blood in EtOh | -80 | 720576 | *Acanthornis magna magna* | 13/04/2022 | -42.1466 | 145.4661 | female | released live and healthy | 73.9 |
| North East | 406947 | ANU-ST10 | Blood in EtOh | -80 | 720576 | *Acanthornis magna magna* | 21/03/2022 | -41.2127 | 148.0162 | female | released live and healthy | 209 |
| North East | 406948 | ANU-ST11 | Blood in EtOh | -80 | 720576 | *Acanthornis magna magna* | 21/03/2022 | -41.2127 | 148.0162 | male | released live and healthy | 158 |
| North East | 406949 | ANU-ST12 | Blood in EtOh | -80 | 720576 | *Acanthornis magna magna* | 21/03/2022 | -41.2127 | 148.0162 | female | released live and healthy | 56.4 |
| North East | 406950 | ANU-ST13 | Blood in EtOh | -80 | 720576 | *Acanthornis magna magna* | 21/03/2022 | -41.2127 | 148.0162 | male | released live and healthy | 140 |
| North East | 406951 | ANU-ST14 | Blood in EtOh | -80 | 720576 | *Acanthornis magna magna* | 22/03/2022 | -41.3214 | 147.925 | male | released live and healthy | 130 |
| North East | 406952 | ANU-ST15 | Blood in EtOh | -80 | 720576 | *Acanthornis magna magna* | 22/03/2022 | -41.3214 | 147.925 | female | released live and healthy | 75.3 |
| North East | 406953 | ANU-ST16 | Blood in EtOh | -80 | 720576 | *Acanthornis magna magna* | 22/03/2022 | -41.3073 | 147.8409 | male | released live and healthy | 37.7 |
| North East | 407009 | ANU-ST10 | Blood in EtOh | -80 | 720576 | *Acanthornis magna magna* | 21/03/2022 | -41.2127 | 148.0162 | female | released live and healthy | 209 |
| South Bruny Island | 406938 | ANU-ST01 | Blood in EtOh | -80 | 720576 | *Acanthornis magna magna* | 14/03/2022 | -43.3902 | 147.3222 | male | released live and healthy | 23.3 |
| South Bruny Island | 406959 | ANU-ST22 | Blood in EtOh | -80 | 720576 | *Acanthornis magna magna* | 28/03/2022 | -43.3838 | 147.3223 | female | released live and healthy | 41.3 |
| South Bruny Island | 406960 | ANU-ST23 | Blood in EtOh | -80 | 720576 | *Acanthornis magna magna* | 28/03/2022 | -43.3896 | 147.2801 | male | released live and healthy | 21 |
| South Bruny Island | 406961 | ANU-ST24 | Blood in EtOh | -80 | 720576 | *Acanthornis magna magna* | 28/03/2022 | -43.3896 | 147.2801 | male | released live and healthy | 96.8 |
| South East | 406941 | ANU-ST04 | Blood in EtOh | -80 | 720576 | *Acanthornis magna magna* | 13/03/2022 | -42.94207 | 147.23883 | male | released live and healthy | 35.1 |
| South East | 406942 | ANU-ST05 | Feather in EtOh | -80 | 720576 | *Acanthornis magna magna* | 13/03/2022 | -42.94207 | 147.23883 | male | released live and healthy | 126 |
| South East | 406943 | ANU-ST06 | Blood in EtOh | -80 | 720576 | *Acanthornis magna magna* | 17/03/2022 | -43.2259 | 146.8769 | male | released live and healthy | 26 |
| South East | 406944 | ANU-ST07 | Blood in EtOh | -80 | 720576 | *Acanthornis magna magna* | 17/03/2022 | -43.2259 | 146.8769 | male | released live and healthy | 76 |
| South East | 406945 | ANU-ST08 | Blood in EtOh | -80 | 720576 | *Acanthornis magna magna* | 17/03/2022 | -43.2204 | 146.8765 | male | released live and healthy | 104 |
| South East | 406946 | ANU-ST09 | Feather in EtOh | -80 | 720576 | *Acanthornis magna magna* | 17/03/2022 | -43.274 | 146.9385 | male | released live and healthy | 171 |
| South East | 406954 | ANU-ST17 | Blood in EtOh | -80 | 720576 | *Acanthornis magna magna* | 24/03/2022 | -42.9448 | 146.7915 | male | released live and healthy | 70.5 |
| South East | 406955 | ANU-ST18 | Blood in EtOh | -80 | 720576 | *Acanthornis magna magna* | 24/03/2022 | -42.9448 | 146.7915 | female | released live and healthy | 41.1 |
| South East | 406956 | ANU-ST19 | Blood in EtOh | -80 | 720576 | *Acanthornis magna magna* | 24/03/2022 | -42.9622 | 146.7839 | male | released live and healthy | 89.8 |
| South East | 406957 | ANU-ST20 | Blood in EtOh | -80 | 720576 | *Acanthornis magna magna* | 24/03/2022 | -42.98 | 146.9993 | female | released live and healthy | 68.3 |
| South East | 406958 | ANU-ST21 | Blood in EtOh | -80 | 720576 | *Acanthornis magna magna* | 24/03/2022 | -42.98 | 146.9993 | male | released live and healthy | 13.6 |
| South East | 407011 | ANU-ST09 | Feather in EtOh | -80 | 720576 | *Acanthornis magna magna* | 17/03/2022 | -43.274 | 146.9385 | male | released live and healthy | 171 |
| South West | 406939 | ANU-ST02 | Blood in EtOh | -80 | 720576 | *Acanthornis magna magna* | 8/03/2022 | -43.36953 | 146.12834 | female | released live and healthy | 16.1 |
| South West | 406940 | ANU-ST03 | Blood in EtOh | -80 | 720576 | *Acanthornis magna magna* | 9/03/2022 | -43.43378 | 146.1817 | male | released live and healthy | 43.7 |
| South West | 407005 | ANU-ST68 | Blood in EtOh | -80 | 720576 | *Acanthornis magna magna* | 16/04/2022 | -43.0373 | 146.3017 | male | released live and healthy | 197 |
| South West | 407006 | ANU-ST69 | Blood in EtOh | -80 | 720576 | *Acanthornis magna magna* | 16/04/2022 | -43.0379 | 146.3016 | male | released live and healthy | 55.1 |
| South West | 407007 | ANU-ST70 | Blood in EtOh | -80 | 720576 | *Acanthornis magna magna* | 16/04/2022 | -43.0373 | 146.3017 | female | released live and healthy | 42.3 |
| South West | 407010 | ANU-ST68 | Blood in EtOh | -80 | 720576 | *Acanthornis magna magna* | 16/04/2022 | -43.0373 | 146.3017 | male | released live and healthy | 197 |
| Tasman Peninsula | 406962 | ANU-ST25 | Blood in EtOh | -80 | 720576 | *Acanthornis magna magna* | 1/04/2022 | -43.1035 | 147.8819 | male | released live and healthy | 13.3 |
| Tasman Peninsula | 406963 | ANU-ST26 | Blood in EtOh | -80 | 720576 | *Acanthornis magna magna* | 1/04/2022 | -43.1048 | 147.8825 | male | released live and healthy | 13.1 |
| Tasman Peninsula | 406964 | ANU-ST27 | Blood in EtOh | -80 | 720576 | *Acanthornis magna magna* | 1/04/2022 | -43.1048 | 147.8825 | female | released live and healthy | 15.4 |
| Tasman Peninsula | 406965 | ANU-ST28 | Blood in EtOh | -80 | 720576 | *Acanthornis magna magna* | 1/04/2022 | -43.1118 | 147.9074 | female | released live and healthy | 4.26 |
| Tasman Peninsula | 406966 | ANU-ST29 | Blood in EtOh | -80 | 720576 | *Acanthornis magna magna* | 1/04/2022 | -43.1118 | 147.9074 | female | released live and healthy | 47.7 |
| Tasman Peninsula | 406967 | ANU-ST30 | Blood in EtOh | -80 | 720576 | *Acanthornis magna magna* | 1/04/2022 | -43.1118 | 147.9074 | male | released live and healthy | 80.5 |
| Tasman Peninsula | 406968 | ANU-ST31 | Blood in EtOh | -80 | 720576 | *Acanthornis magna magna* | 1/04/2022 | -43.1118 | 147.9074 | male | released live and healthy | 58.9 |
| Tasman Peninsula | 406969 | ANU-ST32 | Blood in EtOh | -80 | 720576 | *Acanthornis magna magna* | 1/04/2022 | -43.1094 | 147.9127 | male | released live and healthy | 52.6 |
| South Bruny Island | 406970 | ANU-ST33 | Blood in EtOh | -80 | 720576 | *Acanthornis magna magna* | 5/04/2022 | -43.3564 | 147.2855 | female | released live and healthy | 12 |
| South Bruny Island | 406971 | ANU-ST34 | Blood in EtOh | -80 | 720576 | *Acanthornis magna magna* | 5/04/2022 | -43.3656 | 147.2767 | male | released live and healthy | 40.6 |
| South Bruny Island | 406972 | ANU-ST35 | Blood in EtOh | -80 | 720576 | *Acanthornis magna magna* | 5/04/2022 | -43.3656 | 147.2767 | female | released live and healthy | 10.3 |

**Table S2:** Summary of steps used to filter the VCF file in R.

| **Package** | **Function** | **Criteria** | **Scaffolds** | **Variants** | **Samples** | **% missing data** |
| --- | --- | --- | --- | --- | --- | --- |
|  |  |  | 78369 | 122605 | 94 | 51.54 |
| SNPfiltR | hard_filter | min depth = 5  min genotype quality = 20 | 78369 | 122605 | 94 | 63.63 |
|  | max_depth | max depth = 137 (i.e. 2*SD) | 59557 | 111893 | 94 | 66.41 |
|  | min_mac | minimum minor allele count = 3 | 27819 | 26918 | 94 | 44.94 |
|  | filter_allele_balance | min ratio = 0.2  max ratio = 0.8 | 27819 | 26918 | 94 | 31.33 |
|  | filter_biallelic | keep biallelic SNPs only | 27819 | 26918 | 94 | 31.33 |
| Custom script |  | remove SNPs missing >5% data | 4774 | 9849 | 94 | 1.63 |
|  |  | minimum minor allele count = 3 | 4774 | 7492 | 94 | 1.63 |
|  |  | retain SNPs with Ho < 0.6 | 4770 | 7485 | 94 | 1.63 |
| SNPRelate | snpgdsLDpruning | method="corr", ld.threshold = 0.5, slide.max.bp = 500000, autosome.only = FALSE | 4770 | 5239 | 94 | 1.61 |
| Custom script |  | Remove replicate samples and highly-related individuals | 4770 | 5239 | 70 | 0.98 |
|  |  | Remove sex-linked loci | 4770 | 5239 | 70 | 0.98 |
|  | tess3 & pvalue | Remove loci under putative selection | 4770 | 5239 | 70 | 0.98 |
|  | gl.outflank | Remove loci under putative selection | 4770 | 5239 | 70 | 0.98 |

**Table S3:** Genome assembly statistics of the Tasmanian scrubtit (*Acanthornis magna magna*).

| Metric |  |
| --- | --- |
| Assembly size (Gb) | 1.48 |
| Number of contigs | 1,516 |
| Contig N50 (Mb) | 7.715 |
| Contig L50 | 41 |
| Contig N90 (Mb) | 0.521 |
| Contig L90 | 408 |
| Longest contig (Mb) | 60.595 |
| GC content (%) | 42.88 |
| Complete BUSCOs | 97.1% [Single copy: 96.4%; Duplicated: 0.7%] |
| Fragmented BUSCOs | 0.5% |
| Missing BUSCOs | 2.4% |

**Table S4:** Classification of repeat elements of the Tasmanian scrubtit (*Acanthornis magna magna*) genome assembly.

| **Repeat element** | **Number of elements** | **% of sequence** |
| --- | --- | --- |
| SINEs | 2,575 | 0.03 |
| LINES  LINE1  L3/CR1 | 162,627  416  160,090 | 15.93  0  15.37 |
| LTR elements  ERVL  ERV Class I  ERV Class II | 56,446  30,371  20,5402,917 | 7.79  4.89  2.65  0.16 |
| DNA elements  hAT-Charlie | 17,631  118 | 0.58  0 |
| Unclassified | 133,829 | 6.18 |
| Total interspersed repeats |  | 30.52 |
| Small RNA | 958 | 0.06 |
| Satellites | 25,152 | 1.26 |

**Table S5**: Pairwise F_ST_ values showing genetic differentiation between scrubtit subpopulations. Bootstrapped *p*-value estimates above the are not known as all estimates were <.001. King Island scrubtit subpopulations shown in purple, Tasmanian scrubtit subpopulations shown in yellow.

|  |  | Central North | Central West | Colliers Swamp | Nook | North East | Pegarah | Bruny Island | South East | South West | Tasman Peninsula |
| --- | --- | --- | --- | --- | --- | --- | --- | --- | --- | --- | --- |
| PWF_ST_ | Central North |  |  |  |  |  |  |  |  |  |  |
|  | Central West | 0.01 |  |  |  |  |  |  |  |  |  |
|  | Colliers Swamp | 0.2 | 0.22 |  |  |  |  |  |  |  |  |
|  | Nook | 0.16 | 0.17 | 0.15 |  |  |  |  |  |  |  |
|  | North East | 0.12 | 0.12 | 0.33 | 0.29 |  |  |  |  |  |  |
|  | Pegarah | 0.18 | 0.2 | 0.18 | 0.12 | 0.31 |  |  |  |  |  |
|  | Bruny Island | 0.09 | 0.08 | 0.28 | 0.24 | 0.18 | 0.26 |  |  |  |  |
|  | South East | 0.03 | 0.01 | 0.21 | 0.17 | 0.12 | 0.18 | 0.07 |  |  |  |
|  | South West | 0.02 | 0.01 | 0.23 | 0.18 | 0.13 | 0.21 | 0.07 | <0.01 |  |  |
|  | Tasman Peninsula | 0.23 | 0.24 | 0.43 | 0.39 | 0.22 | 0.41 | 0.27 | 0.22 | 0.24 |  |

**Table S6:** Genetic diversity metrics for a-priori populations of King Island and Tasmanian scrubtits. Shown are mean subpopulation estimates ± the standard errors.

| **Region** | **Locality** | **A** | **A_E_** | **H_E_** | **H_O_** | **II_C_** | **A_R_** | **P_A_** |
| --- | --- | --- | --- | --- | --- | --- | --- | --- |
| King Island | Colliers Swamp | 1.403 ± .006 | 1.245 ± .004 | 0.161 ± .003 | 0.16 ± .003 | 0.342 ± 0.020 | 1.274 | 45.69 ± 0.09 |
|  | Nook | 1.47 ± .006 | 1.288 ± .005 | 0.188 ± .003 | 0.187 ± .003 | 0.251 ± 0.018 | 1.321 | 39.28 ± 0.17 |
|  | Pegarah | 1.431 ± .006 | 1.269 ± .005 | 0.175 ± .003 | 0.156 ± .003 | 0.365 ± 0.048 | 1.298 | 46.77 ± 0.14 |
| Mainland | Central North | 1.806 ± .005 | 1.431 ± .004 | 0.273 ± .002 | 0.256 ± .003 | 0.101 ± 0.009 | 1.481 | 41.33 ± 0.50 |
|  | Central West | 1.738 ± .006 | 1.419± .004 | 0.273 ± .003 | 0.255 ± .003 | 0.093 ± 0.008 | 1.472 | 32.99 ± 0.62 |
|  | North East | 1.597 ± .006 | 1.331 ± .005 | 0.213 ± .003 | 0.205 ± .003 | 0.196 ± 0.007 | 1.37 | 39.05 ± 0.27 |
|  | South Bruny Island | 1.65 ± .006 | 1.38 ± .005 | 0.243 ± .003 | 0.232 ± .003 | 0.170 ± 0.010 | 1.421 | 84.16 ± 0.43 |
|  | South East | 1.824 ± .005 | 1.433 ± .004 | 0.274 ± .002 | 0.259 ± .003 | 0.078 ± 0.009 | 1.484 | 57.85 ± 1.33 |
|  | South West | 1.705 ± .006 | 1.412 ± .005 | 0.274 ± .003 | 0.256 ± .003 | 0.085 ± 0.017 | 1.467 | 29.09 ± 0.42 |
|  | Tasman Peninsula | 1.432 ± .006 | 1.256 ± .004 | 0.161 ± .003 | 0.154 ± .003 | 0.354 ± 0.006 | 1.279 | 65.79 ± 0.34 |

* Denotes 95 % lower and upper confidence intervals

**Table S7:** Annotation of candidate SNPs associated with baldness. For SNPs in non-genic regions, the position and annotation of the closest gene upstream and downstream of the SNP are provided.

| **Genome contig** | **Position of candidate SNP** | **Genic/non-genic** | **Position of gene or closest genes** | **Gene annotation** |
| --- | --- | --- | --- | --- |
| ptg000013 | 11097938 | Non-genic | 11034629-11037505 | *CALHM3*, calcium homeostasis modulator protein 3 |
|  |  |  | 11137523-11196035 | *NEURL1*, neuralised E3 ubiquitin protein ligase 1 |
| ptg000048 | 2661559 | Genic | 2551173-2690658 | *GRIA3*, glutamate ionotropic receptor AMPA type subunit 3 |
| ptg000048 | 4113929 | Genic | 4091125-4123719 | *DOCK11,* dedicator of cytokinesis 11 |
| ptg000048 | 4025573 | Non-genic | 3933269-4012663 | *TNIK*, TRAF2 and NCK interacting kinase |
|  |  |  | 4025614-4034150 | *MAP4K4*, mitogen-activated protein kinase kinase kinase kinase 4 |
| ptg000272 | 649547 | Non-genic | 611019-613166 | *MYH6,* myosin-6 |
|  |  |  | 651350-652559 | Unknown protein |
| ptg000602 | 69459 | Genic | 31541-121032 | *NCOA1*, nuclear receptor coactivator 1 |

**Figure S1a:** Cluster dendrogram showing the bitwise genetic distance between scrubtit samples. Branch labels correspond to the bioplatforms library ID, the dual library index ID and the library index sequence. Labels with R- denote technical replicates. Tight pairs on long branches denote replicated samples, note however that not all replicates are labelled as technical replicates.

**Figure S1b**: Cluster dendrogram showing the bitwise genetic distance between scrubtit samples. Branch labels correspond to the sampling locations. Tight pairs on long branches denote replicated samples as shown in Figure S1a.


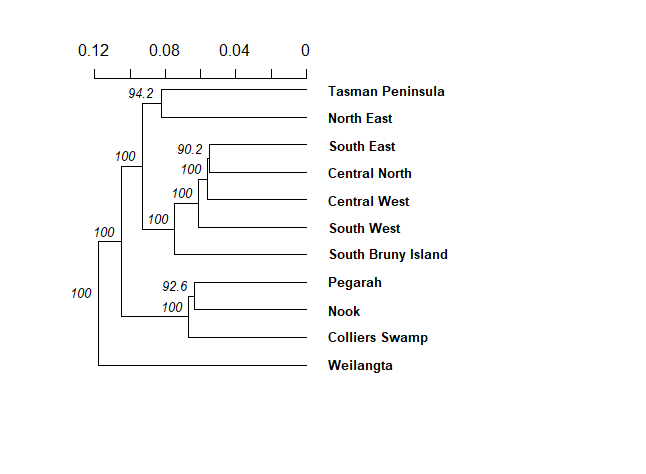


**Figure S2:** Bootstrapped dendrogram showing subpopulation-level differentiation in the King Island and Tasmanian scrubtit. Values on branches denote bootstrapped probability estimates. Weilangta population was just a single sample, so the position of that population is likely to change with additional samples from that locality.


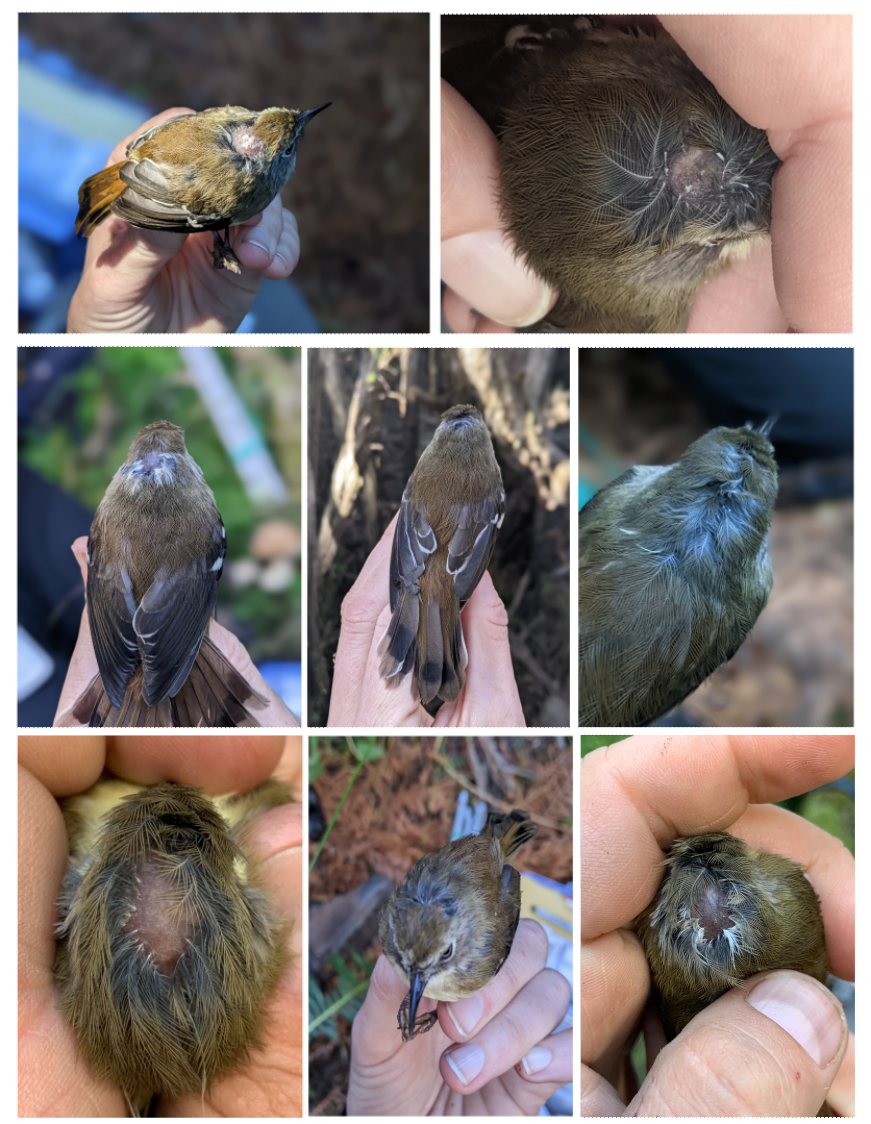


**Figure S3:** Images of crown baldness in affected King Island scrubtits.

*
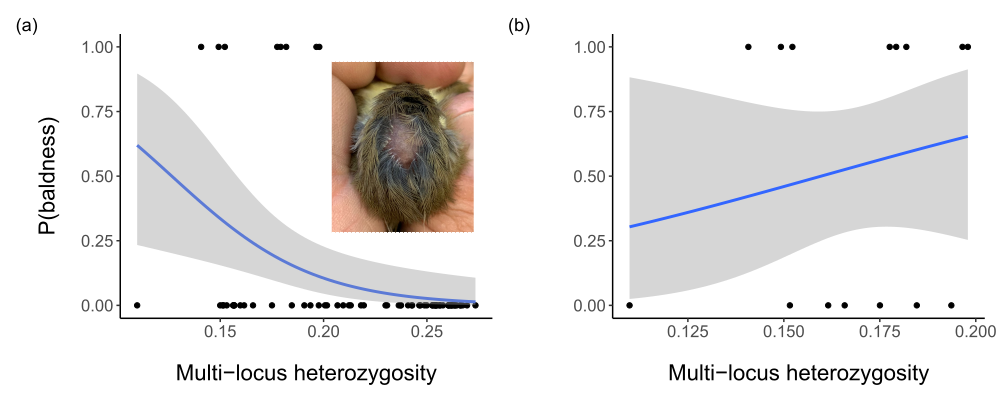
*

**Figure S4:** Logistic regression plots showing the relationship between multi-locus heterozygosity and probability of crown baldness in (a) all scrubtit samples and (b) King Island scrubtits. Inset image within (a) shows example of King Island scrubtit with crown baldness.


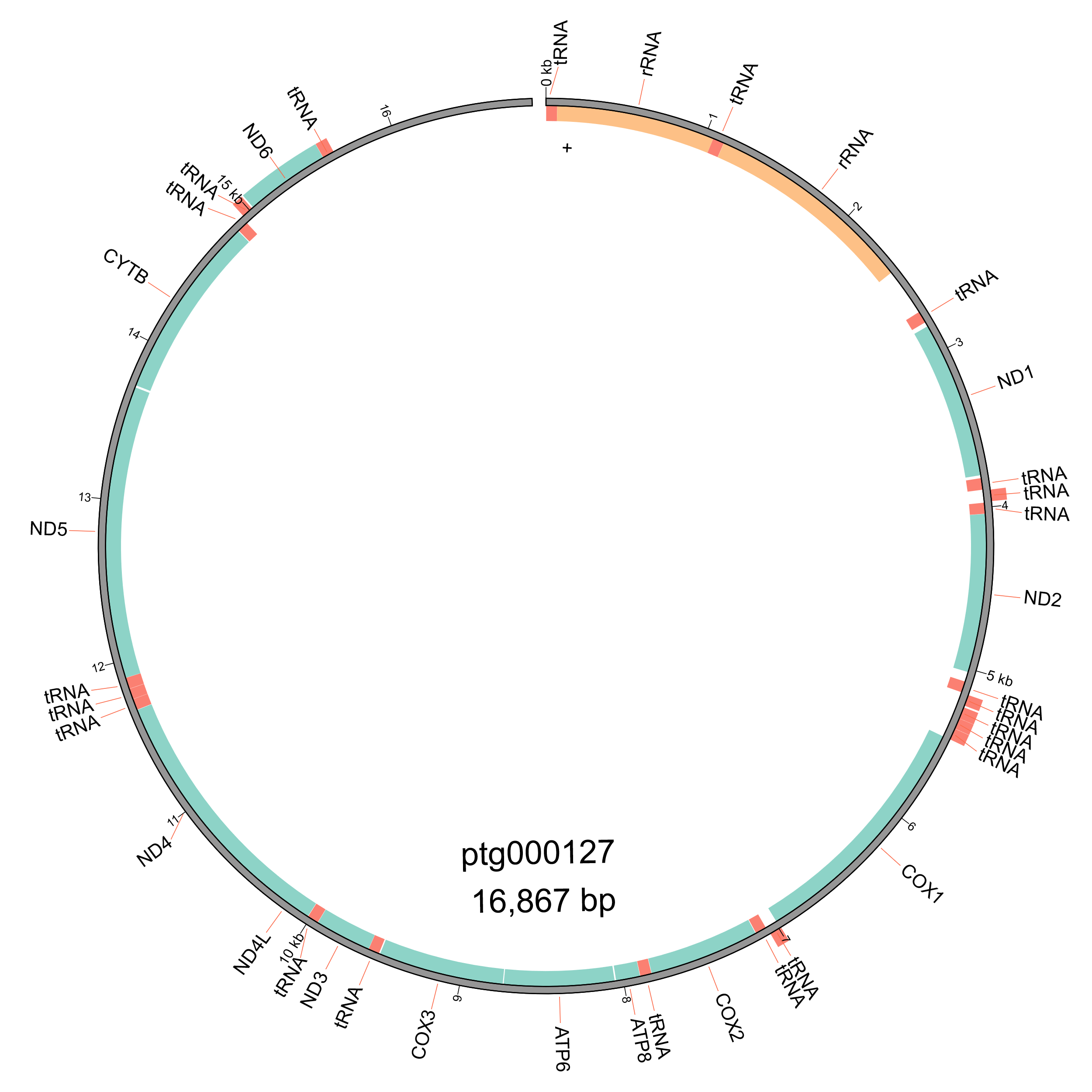


**Figure S5:** Mitochondrial genome of the Tasmanian scrubtit (*Acanthornis magna magna*).


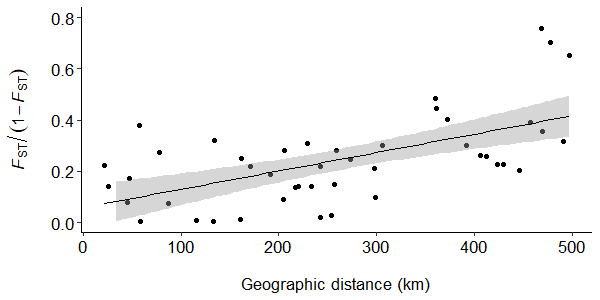


**Figure S6:** Standardized relationship between geographic distance and individual-level genetic differentiation in King Island and Tasmanian scrubtits. Prediction is derived from a linear model, shading represents 95% confidence interval.


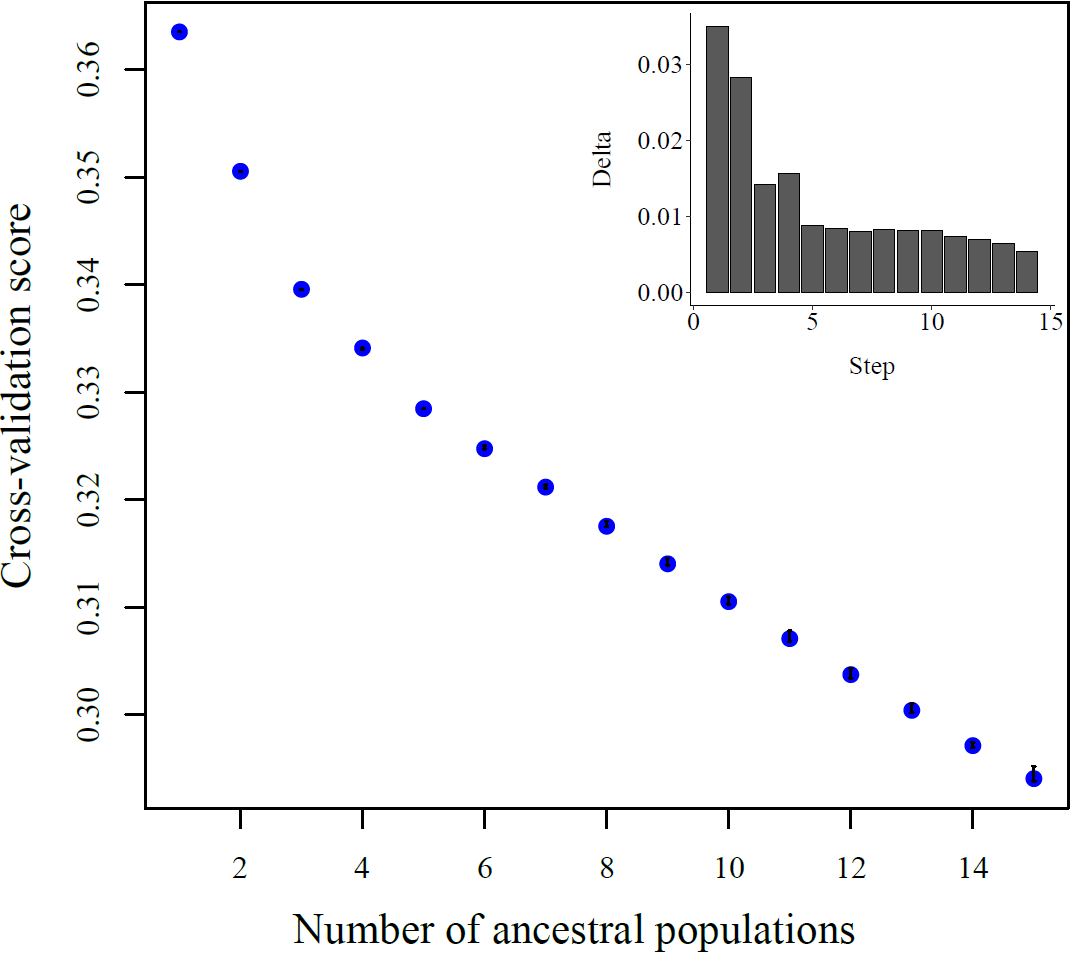


**Figure S7:** Cross-entropy plot used to identify hierarchical population structuring in the genomic dataset for King Island and Tasmanian scrubtits. Lower values of the cross-entropy criterion indicate a better fit to the data. The relatively large drops in cross-entropy scores between 1 and 2, 2 and 3 and (to a lesser extent) between 3 and 4 ancestral populations (*k*) indicates that *k* values of 2, 3 or 4 are well-supported, although higher levels of *k* further subdivide *a-priori* populations into clusters that correspond with the spatial pattern of sampling. The small inset figure shows the absolute change (delta value) in cross-entropy with each step increase in the number of ancestral populations.


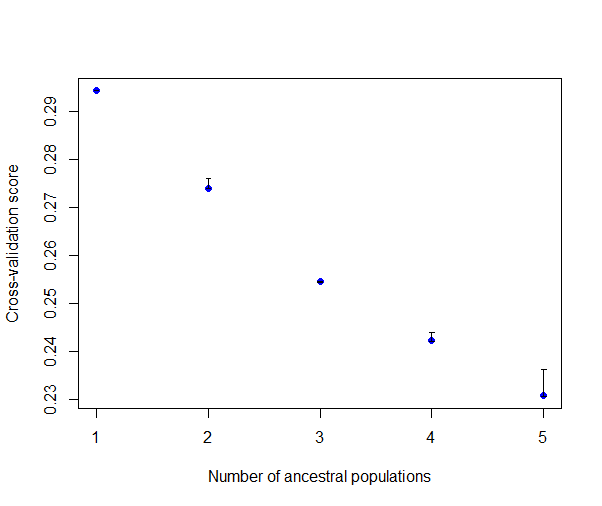


**Figure S8:** Cross-entropy plot used to identify hierarchical population structuring in the genomic dataset for King Island scrubtits. Lower values of the cross-entropy criterion indicate a better fit to the data. The relatively large drops in cross-entropy scores between 1 and 2, 2 and 3 ancestral populations (*k*) indicates that *k* values of 2 and 3 are well-supported.


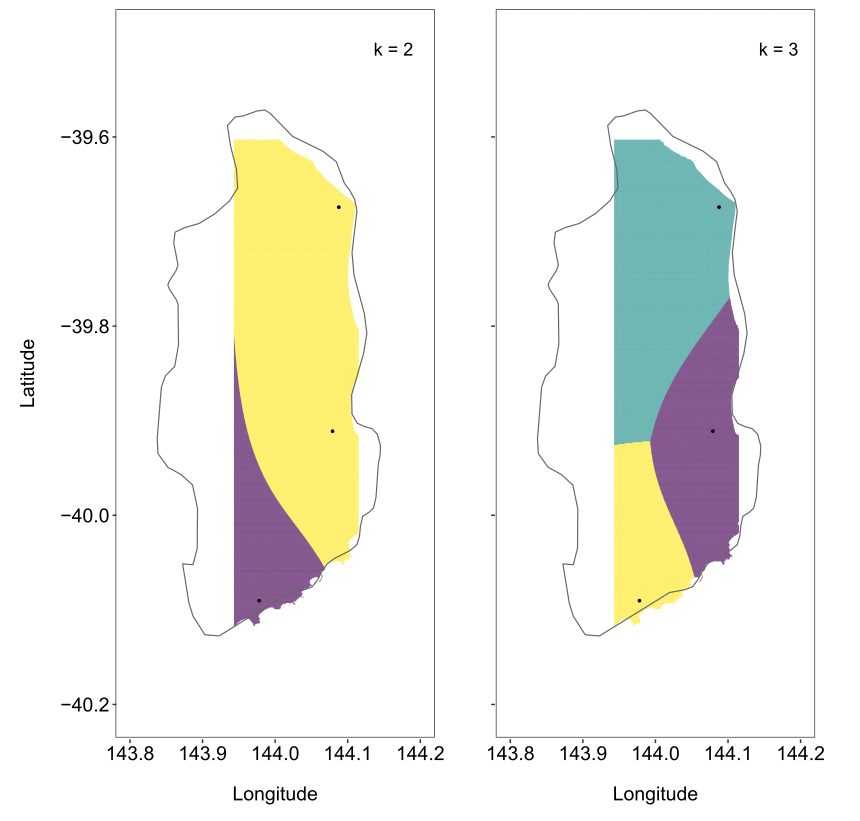


**Figure S9:** Patterns of landscape genomic structure in the King Island scrubtit. Panels show the population genomic structure when two and three ancestral clusters (*k* values) are identified in the data set. Colours in each panel represent the distribution of an ancestral cluster, interpolated across King Island. Black points indicate sampling locations.

*
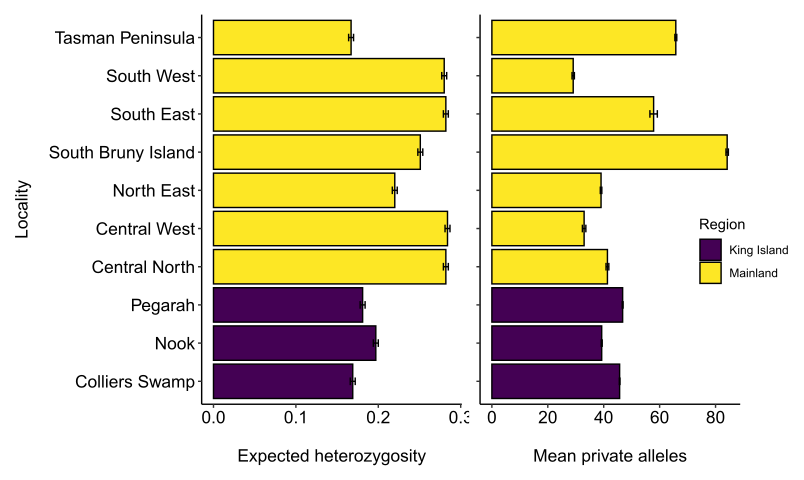
*

**Figure S10:** Estimates of expected heterozygosity and mean private alleles across Tasmanian scrubtit and King Island scrubtit subpopulations. Error bars show standard errors.


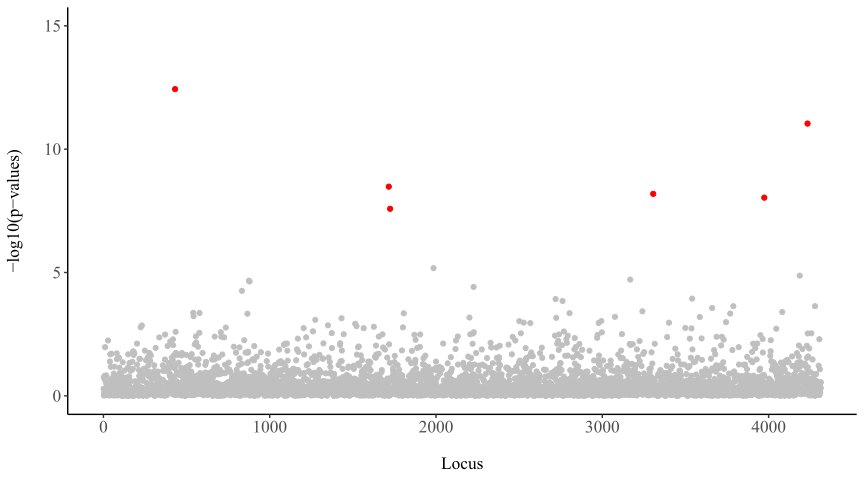


**Figure S11:** Manhattan plot showing the probability of a SNP showing an association with pattern baldness in King Island scrubtits by locus position. The six outlier loci with small *p-*values are shown in red.
